# Mapping football tactical behavior and collective dynamics with artificial intelligence: a systematic review

**DOI:** 10.3389/fspor.2025.1569155

**Published:** 2025-05-30

**Authors:** José E. Teixeira, Eduardo Maio, Pedro Afonso, Samuel Encarnação, Guilherme F. Machado, Ryland Morgans, Tiago M. Barbosa, António M. Monteiro, Pedro Forte, Ricardo Ferraz, Luís Branquinho

**Affiliations:** ^1^ Department of Sports Sciences, Polytechnic of Guarda, Guarda, Portugal; ^2^ Department of Sports Sciences, Polytechnic of Cávado and Ave, Guimarães, Portugal; ^3^ SPRINT—Sport Physical Activity and Health Research & Innovation Center, Guarda, Portugal; ^4^ Research Center in Sports, Health and Human Development, Covilhã, Portugal; ^5^ Research Center for Active Living and Wellbeing (LiveWell), Polytechnic Institute of Bragança, Bragança, Portugal; ^6^ CI-ISCE, Instituto Superior de Ciências Educativas do Douro (ISCE Douro), Penafiel, Portugal; ^7^ Biosciences Higher School of Elvas, Polytechnic Institute of Portalegre, Portalegre, Portugal; ^8^ Life Quality Research Center (LQRC-CIEQV), Santarém, Portugal; ^9^ Department of Sport Sciences, University of Beira Interior, Covilhã, Portugal; ^10^ Department of Sports Sciences, Polytechnic Institute of Bragança, Bragança, Portugal; ^11^ Department of Physical Education, Sport and Human Movement, Universidad Autónoma de Madrid (UAM), Ciudad Universitaria de Cantoblanco, Madrid, Spain; ^12^ Centre of Research and Studies in Soccer (NUPEF), Universidade Federal de Viçosa, Viçosa, Brazil; ^13^ Scientific Department and Department of Athletes' Integration and Development, Paulista Football Federation (FPF), São Paulo, Brazil; ^14^ School of Sport and Health Sciences, Cardiff Metropolitan University, Cardiff, United Kingdom

**Keywords:** performance, tactical analysis, machine learning, neural networks, deep learning, AI

## Abstract

Football, as a dynamic and complex sport, demands an understanding of tactical behaviors to excel in training and competition. Artificial intelligence (AI) has revolutionized the tactical performance analysis in football, offering unprecedented data analytics insights for players, coaches, and analysts. This systematic review aims to examine and map out the current state of research on AI-based tactical behavior, collective dynamics, and movement patterns in football. A total of 2,548 articles were identified following the Preferred Reporting Items for Systematic Reviews and Meta-Analyses guidelines and the Population-Intervention-Comparators-Outcomes framework. By synthesizing findings from 32 studies, this review elucidates the available AI-based techniques to analyze tactical behavior and identify the collective dynamic based on artificial neural networks, deep learning, machine learning, and time-series techniques. Concretely, the tactical behavior was expressed by spatiotemporal tracking data using convolutional neural networks, recurrent neural networks, variational recurrent neural networks, and variational autoencoders, Delaunay method, player rank, hierarchical clustering, logistic regression, XGBoost, random forest classifier, repeated incremental pruning produce error reduction, principal component analysis, and T-distributed stochastic neighbor embedding. Furthermore, collective dynamics and patterns were mapped by graph metrics such as betweenness centrality, eccentricity, efficiency, vulnerability, clustering coefficient, and page rank, expected possession value, pitch control map classifier, computer vision techniques, expected goals, 3D ball trajectories, dangerousity assessment, pass probability model, and total passes attempted. The performance of technical-tactical key indicators was expressed by team possession, team formation, team strategy, team-space control efficiency, determining team formations, coordination patterns, analyzing player interactions, ball trajectories, and pass effectiveness. In conclusion, the AI-based models can effectively reshape the landscape of spatiotemporal tracking data into training and practice routines with real-time decision-making support, performance prediction, match management, tactical-strategic thinking, and training task design. Nevertheless, there are still challenges for the real practical application of AI-based techniques, as well as ethical regulation and the formation of professional profiles that combine sports science, data analytics, computer science, and coaching expertise.

## 1 Introduction

Football has been described as a complex, dynamic, and non-linear system, in which the confrontation of two teams depends on constant adaptation to technical-tactical actions, situational factors, and ever-changing game situations (1, 2). A football team's performance and success depend on a deep comprehension of collective behavior, encompassing everything from individual player movements to the interdependence between game model, strategy, and opposing systems (3, 4). However, the practical operationalization of all the dimensions that influence tactical behavior and patterns has led to the development of complex and time-consuming methodologies, highly dependent on experience and susceptible to human error (3, 5). Although the automation of information collection systems such as tracking systems based on global position system (GPS) or Global Navigation Satellite Systems, local position measure (LPM), or video-based motion analysis (VBMA) has been already a widespread procedure in technical teams (6–8), the quantification of this information, the visualization datasets, and the dynamics of the work teams have undergone some transformation in recent years (9, 10).

In recent years, the integration of artificial intelligence (AI) techniques has revolutionized the analysis of tactical behaviors in football, offering unparalleled insights and opportunities for enhancement across various facets of the game. Data science and data analytics departments have been springing up in football clubs, exploring data analysis routines and procedures that normally applied IA techniques such as artificial neural networks (ANNs), deep learning (DL), and machine learning (ML) (2, 11). All these procedures require advanced computing environments and can be developed using supervised or unsupervised trainable algorithms (12, 13). Typically, this type of analysis is based on two datasets: spatiotemporal (14, 15) and key performance indicators (KPIs) (16, 17). On the one hand, spatiotemporal data are based on the time-series analysis (TS) raw data (*x, y, z*) of the individual and collective positions that the tracking systems provide. On the other hand, KPIs are based on notational and observational analysis, major areas of match analysis 1.0 and 2.0, which allow performance to be assessed in individual and collective actions (3, 18). All these datasets have been gaining ground in an integrative view of all football performance dimensions, especially physical, physiological (9, 19), and technical-tactical factors (20–22). The integrative view of the data allows us to better understand the preponderant factors in the interdependence of the match-related factors, intra- and intercoordination team formation, playing style, or tactical-strategic nuances (4, 21, 23).

The integration of AI in football analysis has ushered in a new era of understanding, enabling players, coaches, and performance analysts to glean deeper insights into big data (11, 13). With advancements in technology, researchers and practitioners can now decipher patterns and trends that were previously inaccessible, thereby informing accurately the decision-making processes during training and competition (11, 13, 24). Moreover, AI-based tactical behavior mapping holds immense promise in enhancing player development, refining coaching strategies, and elevating the overall standard of match analysis (3, 5). However, a comprehensive overview of football analytics remains to be established in the literature, which would allow for an in-depth examination of the intersection between AI and tactical behavior mapping (11, 13, 24). Football matches can be evaluated physiologically, technically, and tactically in a dynamic manner (match analysis level 3.0–4.0) with the use of spatiotemporal data (25).

Through a comprehensive evaluation of existing literature, this systematic review endeavors to identify the strengths and limitations of current approaches, while also illuminating avenues for future research and technological advancements in this burgeoning field (4, 26). By synthesizing disparate findings and insights, this review aims to provide a comprehensive understanding of the AI's role in augmenting tactical awareness and optimizing performance in football (11, 13, 24). Thus, this systematic review aims to examine and map out the current state of research on AI-based tactical behavior, collective dynamics, and movement patterns in football. By synthesizing findings from a diverse range of studies, this review seeks to shed light on the methodologies, technologies, and outcomes employed in the analysis of tactical behaviors within the realm of football. Specifically, it explores the utilization of neural networks, ML algorithms, computer vision techniques, and data analytics frameworks for extracting actionable insights from player movements, team formations, and strategic decision-making processes.

## 2 Materials and methods

### 2.1 Literature search strategy

The Preferred Reporting Items for Systematic Reviews and Meta-Analyses (PRISMA) guidelines and the Population-Intervention-Comparators-Outcomes (PICOS) design were followed to conduct this systematic review (27, 28). The literature search was based on seven academic databases and digital libraries: Web of Science (WoS, including all Web of Science Core Collection: Citation Indexes), PubMed/Medline, Science Direct (SCOPUS), SportDiscus, ACM Digital Library, IEEE Xplore Digital Library, and arXiv.org (e-Print archive). The eligibility criteria were established following the PICOS approach, and the search strategy was defined as follows: (1) Population: adult and youth football players (≥14 years old); (2) Intervention: AI-based analysis of offensive and defensive football patterns; (3) Comparison: AI techniques (ML, DL, neural networks); (4) Outcomes: tactical behavior and collective dynamics; (5) Study design: experimental and quasi-experimental designs. In accordance with the search strategy, studies from January 2000 to April 2025 were included for relevant publications using keywords presented in Table 1. In addition, the study variables are a Boolean search phrase (Table 1).

**Table 1:** Search terms and following keywords for screening procedures.

Search term	Keywords
1 Football (population)	*(“football” OR “soccer”)*
2 Artificial intelligence (dependent variable)	*(“artificial intelligence” OR “machine learning” OR “neural networks” OR “deep learning” OR “data analytics”)*
3 Tactical analysis (independent variables)	*(“match analysis” OR “tactical analysis” OR “collective behaviour” OR “team behavior” OR “tactical patterns” OR “offensive” OR “defensive” and “team formation” OR “tactical performance” OR “performance analysis” OR “technical-tactical performance”)*
4 Boolean search phrase (final search)	*1 AND (2 OR 3)*

The literature search was accessed between January and March 2025. The search strategy was independently conducted by one review author and checked by a second author. Discrepancies between the authors in the study selection were solved with support of a third reviewer. The authors did not prioritize authors or journals.

### 2.2 Selection criteria

The included studies in the present review followed the subsequent inclusion criteria: (1) Studies applying AI algorithms and techniques to analyze behavior and tactical patterns in football from both sexes of adult, youth competitions; (2) studies with screening procedures based on ANN, DL, ML, TS, and technical-tactical KPI; (3) only studies that included the AI-based method to express tactical analysis (i.e., team formation, style, patterns, networks); (4) observational prospective cohort, case-control, and/or cross sectorial design study including with at least 1-game datasets; (5) studies of human physical and physiological performance in Sport Science and as scope; (6) original article published in a peer-review journal; (7) full text available in English; and (8) article reported sample and screening procedures (e.g., data collection, study design, instruments, and outcomes).

The exclusion criteria were as follows: (1) Studies applying AI algorithms and techniques to other key outcomes in football (e.g., predict match outcome, player selection, injury prevention, isolated physical/physiological performance); (2) studies without screening procedures based on specifically ANN, DL, ML, TS, and KPI; (3) applying spatiotemporal data or KPI without AI-based analysis to map out tactical behavior, collective dynamics and movement patterns; (4) AI-based studies in other football codes (i.e., Rugby, Australian Football, Gaelic Football, and Beach Football, Futsal); (5) other research areas and non-human participants; (6) articles with bad quality in the description of study sample and screening procedures (e.g., data collection, study design, instruments, and the measures) according to Downs and Black scale; (7) reviews, abstract/papers conference, surveys, opinion pieces, commentaries, books, periodicals, editorials, case studies, non-peer-reviewed text, masters, and/or doctoral thesis.

Up until April 2025, only original articles published online could be found with the search. First, titles and abstracts were chosen and rejected based on the predetermined criteria. The selection process used to establish the final status—inclusion or exclusion—was applied to full-text articles. Arguments were settled by dialogue between the two authors, or, if needed, by a third researcher. There are now additional pertinent secondary sources that went through the same screening processes.

### 2.3 Quality assessment

Following the PRISMA statement, a systematic search of relevant English-language articles was performed between 2000 and 2025 (27, 28). The methodological quality of the studies was evaluated using the modified Downs and Black Quality Index (29), comprising 14 items. Higher scores indicated higher-quality studies, with scores above 0.6 considered indicative of a superior methodological quality. The quality statement (QS) conducted with an interobserver reliability analysis was conducted afterward, and each author carried out the classification on their own (Kappa index: 0.96).

### 2.4 Study coding and data extraction

**Figure 1:**
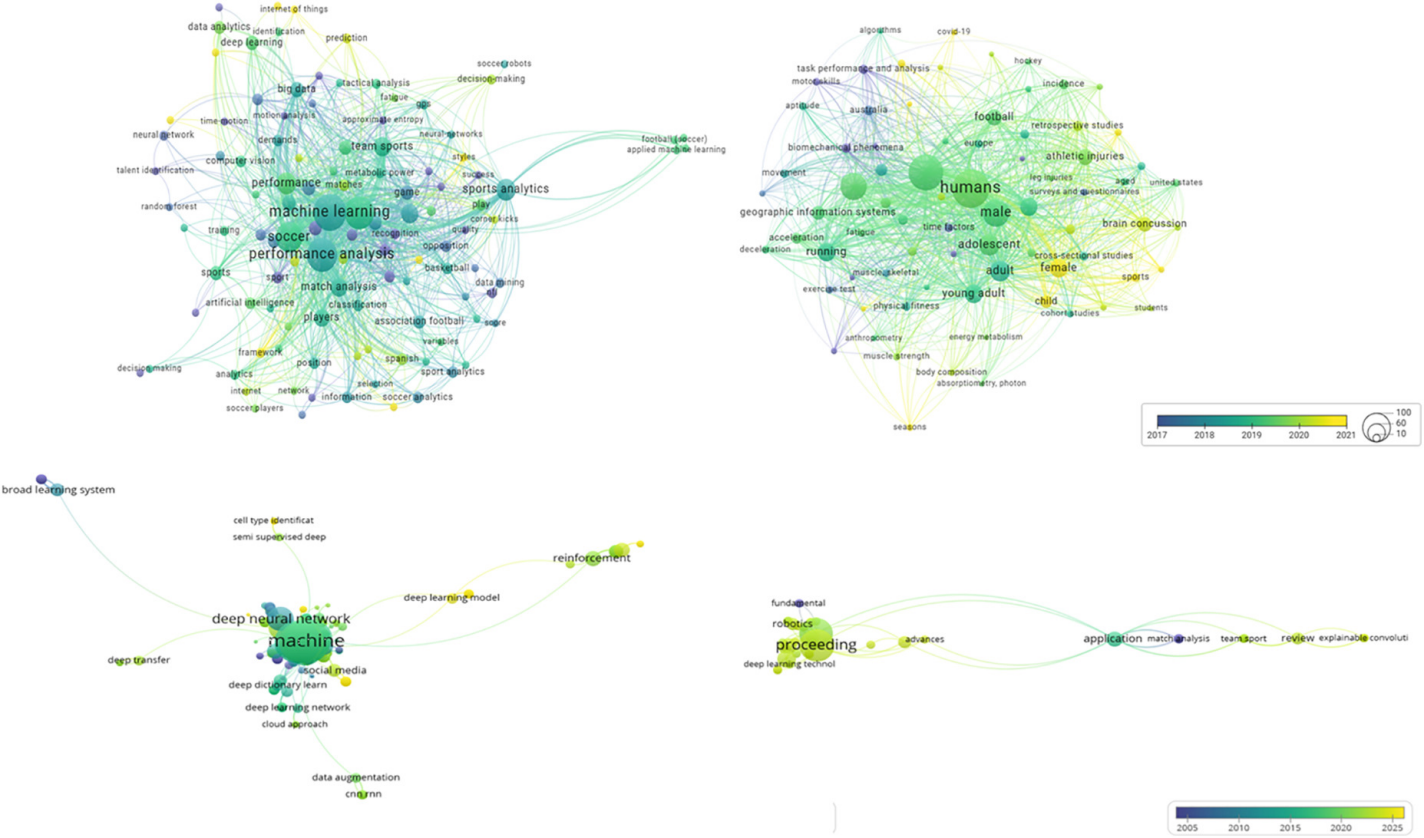
Most recurrent keywords in WoS and sportDiscuss collections. Different years, which facilitates temporal analysis. Hot research topics are represented by different colors in the graph of the average year of publication by the VOSviewer software with bibliometric occurrence map based on reference management files in specific API requests and search queries from WoS and SportDiscuss, Science Direct, PubMed; IEEE and ACM and arXiv. ACM, ACM Digital Library; AI, Artificial Intelligence; arXiv, arXiv.org (e-Print archive); IEEE, IEEE Transactions on Knowledge and Data Engineering; WoS, Web of Science (Core Collection: Citation Indexes).

Data extractions from the included articles were performed according to the following summary measures (1): (i) AI category; (ii) measures; (iii) formulas; (iv) description; (v) supervision; (vi) training algorithm; (v) accuracy; (vi) reference (Table 2); (2) sample characteristics were described according to: (i) reference; (ii) dataset; (iii) season competition; (iv) sample (*n*); (v) sampling; (vi) platform; (vii) publisher; (viii) quality statement (Table 3); (3) main findings: (i) reference; (ii) dataset; (iii) season competition; (iv) sample (n); (v) sampling; (vi) platform; (vii) publisher; (viii) QS score (Table 4). The research hot topics refers were determined to cover the frequently occurring keywords identified through a bibliometric analysis using VOSviewer software (30), which clustered key AI-based themes from the included studies (Figure 1).

**Table 2:** Summary of measure, formula, description, and AI-based procedures of the reviewed articles.

AI	Measures	Formulas	Description	Supervision	Training algorithm	Accuracy	Reference
ANN DL
	CNN LSTM RNN VRNN VAE	$Z_{i,j}=\;\overset{KH^{-1}}{\underset{m=0}{\sum }}\overset{KH^{-1}}{\underset{m=0}{\sum }}\overset{C-1}{\underset{k=0}{\sum }}X_{(iS+m),(\;jS+n),k\;}\cdot K_{m,n,k}$	Eigenvalue at position (*i*, *j*) is denoted as Z*i*, *j*, where the convolution kernel's height and breadth are represented by *m* and *n*. Each position (*i*S + *m*), (*j*S + *n*) in the input feature map is symbolized by X (*i*S + *m*), (*j*S + *n*), *k*, indicating the channel *k*. The weight of the channel at position (*m*, *n*) inside the convolution kernel is labeled as *K*_m_, *n*, *k*.	Unsupervised	ND	88−94%	Wang and Guo (71), Shen et al. (37), Shokrollahi et al. (49)
		$Y_{i}=\max_{l,t}\cdot X_{(iS+l),(\;jS+t),k}$	ReLU activation function: *Y_i_*, *j*, and *k* represent the eigenvalue at position (*i, j*) in the pooling operation. *t* represents the indices on the height and width of the pooled window, respectively.	Unsupervised	ND	88%	Wang and Guo (71), Shen et al. (37), Shokrollahi et al. (49)
		$Y_{i}=\overset{D-1}{\underset{r=0}{\sum }}X_{r}\cdot W_{i,r}+M$	Fully connected layer: position *i* is represented by *Y_i_*. The neuron index of the layer before is indicated by *r*. *r* in the previous layer's output feature vector is represented by *X_r_*. The weight of linking the *i*-output neuron to the *r* input feature is represented by *W_i_*, *r*, *M* represents the bias of the output neuron.	Unsupervised	ND	88%	Wang and Guo (71), Shen et al. (37), Shokrollahi et al. (49)
	Delaunay method	$A_{ij}(T)=\;\{\begin{array}{c} 1\\ 0 \end{array}$	Voronoi regions of players *i* and *j* are adjacent. Coarse-grained formations for Nc = 3 in the normalized coordinates where the direction of offense is upward.	ND	ND	ND	Narizuka and Yamazaki (55)
	ITFPS SDR	$Similarity_{\;position}\;(Coach,\;Algorithm)=\;\{\begin{array}{c} 1;\;\;coach_{\;position\;\neq }\;ITFPS_{\;position\;}\\ \;0;\;\;coach_{\;position\;\neq }\;ITFPS_{\;position\;} \end{array}$	A greater denominator suggests that the coach successfully chose the right players for the game, and a higher ratio measure shows that the coach placed the chosen players in their proper spots. This score offers a thorough evaluation of how well the coach aligns player positions and selections.	ND	MSE: 80%	ND	Nouraie et al. (72)
		$Accordance_{\;position}\;(Coach,\;Algorithm)=\;\{\begin{array}{c} 1;\;\;coach_{\;position\;\neq }\;Candidates\;set_{\;position\;}\\ \;0;\;\;coach_{\;position\;\neq }\;Candidates\;set_{\;position\;} \end{array}$					
		$Ration=\;\frac{\sum_{\;position}Accordance_{\;position}(Coach,\;Algorithm)}{Knowledge_{coach}}$					
	PlayeRank	$r=(u,m)=\;\frac{1}{R}\overset{n}{\underset{i=1}{\sum }}w_{i}\cdot x_{i}$	*r* (*u*, *m*) is called the performance rating of *u* in match *m*, where *R* is a normalization constant such that *r* (*u*, *m*) ∈ [0, 1].	Unupervisioned	40%	71%	Pappalardo et al. (48)
	Multilayer ANN	$in=\overset{m}{\underset{i=1}{\sum }}(x_{i}\cdot w_{i}+B)$	Gedeon's relative importance calculation: *w* is a set of weights, and each individual input is *x_i_*, and corresponding weight is *w_i_* (*w_i_* and *w*, *x_i_* and *X*).	Unupervisioned	44.16%	81−87%	Yücebaş (56)
	Classification passes	$\emptyset_{j}\;:\;Q\;\times \;\mathrm{Tr}\;\times \;E\;\times \;L\;\to R\;.$	An optimal parameterization, denoted as *θ*, for a classifier function *h_θ_*, which aims to produce output labels close to the ground truth. Each algorithm employs a cost function *J* (*X*, *y*, *θ*).	Supervisioned	$h_{\emptyset }\;:R_{b}\to \;Y$ $\emptyset =\;argmin_{\emptyset }\;J(X,y,\emptyset )$	93%	Chawla et al. (32)
	Player motion	$g(x,\;t_{\;pj}\;,\;s_{i})\;=\;t$	The motion model, denoted as *g*, determines the time *t* ∈ *R* + *it* would take for player *p_j_* to reach a point×∈ *R*2 at time step s*i*, based on the player's trajectory *t_pj_*. This model extrapolates the player's direction and velocity from trajectory at time step s*i* (circle, ellipse, data-driven).	Supervisioned	SVM; MLR; *ε*-SVC RUSBoost	90.2	Chawla et al. (32)
	Entropy clustering	$N=\sum_{n=1}^{N}P\;(x|n)\;P\;(n)=\frac{1}{N}\overset{N}{\underset{\;n=\;1}{\sum }}P_{n}\;(x)$	*D* is to estimate the team's underlying formation, represented by the most probable set *F*^∗^ of 2D probability density functions. Initially, we consider the 2D probability density function *P* (*X* = *n*) to model the tracking data, representing the heatmap.	Unsupervised	EM k-means	ND	Bialkowski et al. (34), Stival et al. (70)
ML	Hierarchical clustering	$h\;(C_{1},C_{2})=\;\frac{2n_{1}n_{2}}{n_{1}+n_{2}}\underset{t_{1}\in C_{1}}{\sum }A(t_{1})-\frac{1}{n_{2}}\underset{t_{2}\in C_{2}}{\sum }A(t_{2}{)}^{2}$	Hierarchical clustering is performed using Ward's method, where the input to the clustering is the dissimilarity matrix *D* whose components are Dtt.	Unsupervised	ND	ND	Narizuka and Yamazaki (55)
	TAI	$TAI\left[\begin{array}{cc} T_{11} & T_{12}\\ T_{21} & T_{22} \end{array}\right]$ (…) ($TAI\;=\;A_{C1}-A_{C2}$ (…) $A_{C1}\;=\;\epsilon \;\times \;(T_{11}+T_{21}-T_{12})$ $A_{C1}\;=\;\epsilon \;\times \;(T_{11}+T_{21}-T_{12})$	To quantify the interaction in a segment, the matrix *M* is further divided into four blocks by summing all the elements (*M i*,*j*) in top left [team-1 ∀ *i*, *j* in (1, …, 14)], and the bottom right [team-2 ∀ *i*, *j* in (15, …, 28)], which represent the overall activity of each team that is obtained by summing the activity of all players in a team.	Unsupervised	SVM	ND	Kusmakar et al. (47)
	Sen	$H=\;-\;\overset{N}{\underset{i=1}{\sum }}p_{i}\ln(\;p_{i})$	*p_i_* is the probability of the *i*-th element in the sequence	Unsupervised	SVM	ND	Kusmakar et al. (47)
	KC	$h\;(N)=\frac{c(N)}{b(N)}$	*b* (*N*) = *N* log_2_ *N*	Unsupervised	SVM	ND	Kusmakar et al. (47)
	DistEn	$DisEn\;(m,\tau ,\;\beta )=\;\frac{1}{\log_{2}(\beta )}\overset{\beta }{\underset{i=1}{\sum }}p_{i}\log_{2}(p_{i})$	*β* = 64 is the number of bins in the probability distribution, obtained from the data with the lag *τ* = 1 and embedding dimension *m* = 2.	Unsupervised	SVM	ND	Kusmakar et al. (47)
	QTC	MPOs (*t_x_* – *t_y_*)	MPOs are depicted through relative movements using QTC characters, detailing the interaction between pairs of MPOs within a time interval. Pairwise relations are stored in a QTC matrix, while sequences of relative movements across time intervals are represented by QTC matrices.	Supervisioned	ND	*k* = 4	Beernaerts et al. (36)
	SoccerMix	$P(x)=\overset{k}{\underset{\;j=\;1}{\sum }}\;\partial j\;\cdot F_{j}(x|\theta_{j})\;$	In the mixture model, $F_{j}$ is a probability distribution or density parameterized by $\theta_{j}$ for the *j*th component, *k* is the number of components, and *α_j_* is the probability of the *j*th component. Mixture models make intuitive sense as a soft clustering variation of k-means clustering.	ND	ND	ND	Decroos et al. (51)
	EGV	$x_{play}=vec(\left[x_{offsensive};x_{defensive}\right]).$	Player's trajectories as a vector of their *x*, *y* position for the full 10 s. As the tracking data is sampled at 10 fps, the dimension of each player's trajectory	Supervisioned	*ND*	ND	Gu et al. (43), Fernando et al. (59)
	LR	$P\;(y=1x)=\;\frac{1}{1+e^{-(w^{T}x+b)}}$	*P* is the probability that the output *y* is 1 given the input *x*; *w* is the weight vector. *b* is the bias term. *x* is the input vector.	Supervisioned	*ND*	ND	Forcher et al. (52)
	XGBoost	$y_{i}=\overset{K}{\underset{k=1}{\sum }}f_{k}(x_{i)}$	*i* is the predicted output for the *i* th instance. KK is the total number of trees. $f_{k}(x_{i)}$) is the prediction of the *k*-th tree on the *i*-th instance.	Supervisioned	*ND*	ND	Forcher et al. (52)
	RF classifier	*ND*	Random forest is an ensemble learning method that constructs a multitude of decision trees at training time and outputs the class that is the mode of the classes (classification) or mean prediction (regression) of the individual trees.	Supervisioned	*ND*	ND	Forcher et al. (52)
	RIPPER	t-SNE	Maintaining pairwise similarities between data points in a lower-dimensional space is the main goal of the nonlinear *t*-SNE method. However, PCA concentrates on maintaining large pairwise distances.	Supervisioned	ND	94.14%	García-Aliaga et al. (38, 53)
		UMAP	The UMAP dimension reduction method can be applied to general non-linear dimension reduction as well as SNE visualization.	Supervisioned	ND	94.14%	García-Aliaga et al. (38)
TS	Betweenness centrality	$c_{Bn}(v)=\underset{s,t\epsilon V}{\sum }\frac{\sigma (s,t|v)}{\sigma (s,t)}$	*σ* (*s*, *t*|*v*) over all distinct pairs of vertices *s*, *t* divided by the total number of shortest paths	ND	ND	ND	Stival et al. (70)
	Eccentricity	$e(v)=\max(d(v,v_{j})),\forall v_{j}\in V$	The distance *d* (*u*, *v*) between two vertices *u*, *v* is defined as the length of the shortest path between *u* and *v*.	ND	ND	ND	Stival et al. (70)
	Efficiency	$Global\;Efficiency:E(v)=\frac{\sum_{\;j\neq v}^{\;j\epsilon G}\frac{1}{d(v,j)}}{\left(n-1\right)}$ $Local\;Efficiency:E_{loc}(v)=\frac{1}{\deg(v)}\underset{vj\in G_{v}}{\sum }E(v_{j}$	Vertex *v* measures the information transmission capacity of the graph through *v* and indicates how efficient it is to send information among vertices. Local efficiency of a vertex *v* is computed for a subgraph *G_v_* that contains the neighbors of v and their edges	ND	ND	ND	Stival et al. (70)
	Vulnerability	$v(v)=1-\frac{E_{loc\;(v)}}{E(v)}$	Vulnerability of a vertex *v* uses global efficiency and the local efficiency of v to compute the drop in efficiency after the *v* removal	ND	ND	ND	Stival et al. (70)
	Clustering coefficient	$C(v)=\frac{2T(v)}{\deg(v)(\deg(v)-1)}$	Clustering coefficient characterizes the presence of triangles (loops of order 3) formed with a vertex *v*. Let *T*(*v*) be the number of triangles formed with *v*.	ND	ND	ND	Stival et al. (70)
	Page rank	$p\;(i)=\;\frac{1-q}{n}+\underset{\;j\in nei(v)}{\sum }\frac{\;p(j)}{\deg(j)}$q	PageRank measures the importance of a vertex *v* using as a source of information the importance of its neighbors. Let *v* be anode in the graph G and *nei*(*v*) be the subset of all neighbours of *v*.	ND	ND	ND	Stival et al. (70)
	DT model	DT, GB, LDA and QDA Classifiers	Classifier performance was evaluated based on mean accuracy score, recall, and precision in predicted wins (given that wins are our focus), and mean Brier score over a fivefold cross-validation.	Supervisioned	ND	74.1%	Goes et al. (39, 42)
	Pitch control map classifier	$p\;(k|\sigma ,\;\lambda ,\;x)=\;\{\begin{array}{c} 1-p\;\;for\;k=0\\ \;p\;for\;k=1 \end{array}$	Bernoulli trial and *k* represent the outcome of the pass with success = 1 and failure = 0. Whether the pass will be successful or not is derived from the input x and two trained parameters σ and *λ*.	Supervisioned	10% dataset, 20,000 sequences in total	85%	Gu et al. (43)
	EPV	$P(x)=\sum_{i=1}^{m}\sum_{\;j=1}^{n}EPV_{ij}\cdot a_{tk}^{ij}$	EPV_ij_ and the possibility of the attacking team controlling the ball, a_ij_ t_k_. The sum of the product of these two values represents the expected possession value for the Pitch Control map.	Supervisioned	10% dataset, 20,000 sequences in total	85%	Gu et al. (43)
	DTMC	$P\;(sns\;|f_{i})=c_{\;fi,sns}|c_{\;fi}$	*c*_fi, sns_ is the number of unsuccessful ball-moving actions from state f_i_, c_fi_,sns is the number of unsuccessful shots from f_i_, c_fi,g_ is the number of goals scored from *fi*, and *c*_fi_ is the total number of actions initiated in *f_i_*.	Unsupervised	ND	ND	Clijmans et al. (31)
	EPS HMM	$f\;(y)=\;\frac{\beta^{\alpha }y^{\alpha -1}e^{-\beta y}}{\Gamma (\alpha )}$	*N* and the class(es) of state-dependent distribution(s) must be selected. Since *y_t_* (i.e., EPS) is strictly positive and continuous, a gamma distribution here [α > 0 and β > 0, and Γ(α) is the gamma function].	Unsupervised	ND	ND	Ötting and Karlis (73)
	Heatmaps	$N=\overset{\infty }{\underset{k=\;0}{\sum }}Q^{k}=(I-Q{)}^{-1}$	*i* is the identity matrix and each entry q*_ij_* of Q is the transition probability from a transient state i to a transient state j. Each entry n*_ij_* is equal to the expected number of visits to a transient state j when starting the possession sequence from state *i*.	Unsupervised	ND	ND	N/A
		B = N R	*N* is the fundamental matrix and R the matrix containing all the transition probabilities from transient to absorbing states.	Unsupervised	ND	ND	N/A
		$M_{i,j}=\frac{1}{\left|H_{i}||H_{j}\right|}\underset{h_{x,}h_{y}\in (H_{j},H_{j})}{\sum }|\left|H_{ix}-H_{y}\right||$	Hi is the set of all the heatmaps constructed for a team *i*. Recall that we partitioned the set of all passes attempted during a season for a single team into 10 random subsets, i.e., |Hi | = 10, ∀ *i*. Each *i*, *j* entry, where *i* = *j*, is the average distance between each of the 10 heatmaps.	Unsupervised	ND	ND	Kim et al. (46)
		$\arg_{W}^{max}Tr\left(\frac{W\sum_{b}W}{W\sum_{\prime }W}\right)$	*Σ*b and *Σ*w are the between-scatter and within-scatter matrices respectively. To test the various representations, only identity experiments on home performances were tested.	Unsupervised	ND	ND	Lucey et al. (45)
KPI	Computer vision	$L\;=\;\{(\;p_{j},\;m_{v,}l_{w}):\;p_{j}\;\in \;P,\;m_{v,}\in \;M,\;l_{w}\;\in \{\mathrm{Home},\;\mathrm{Aw}\;a\;y\;\},\;(\;p_{j},\;m_{v,})\to \;l_{w}\}.$	Lineup L as a mapping from player to team for a match as the trajectory and event data are modeled as sequences, indexed by the global time sequence s. A trajectory *tp* is a sequence of two-dimensional coordinate locations for a particular player *p*. Let Tr be the set of all trajectories.	Unsupervised	ND	ND	Chawla et al. (32)
	xG ECOM EVG	$v_{s}(\;pass_{i})=\frac{\sum_{\;j=\;1}^{l}xG(\;pass\;j)}{l}$	V is the score for the record containing a single pass. The idea is that a pass holds a value that is contingent upon its distance and a weighted average of similar passes, less the average outcome of other passes. K has been deduced from cluster validation and the formative formulas of the metric and pass-xG comparison can be computed.	Supervisioned	$\overset{k}{\underset{i=\;1}{k=\sum }}\overset{N}{\underset{\;n=\;1}{\sum }}||x_{i}^{\left(j\right)}-centroid_{j}|{|}^{2}$	87%	Bravo et al. (61)
	3D ball trajectories	$p=\;\frac{\sum_{i=1}^{X}\sum_{\;j=1}^{Y}(\left(I_{i,j}-m)\cdot (M_{i,j}-m\right))}{\sqrt{\sum_{i=1}^{X}\sum_{\;j=1}^{Y}((I_{i,j}-m)\cdot {\left(M_{i,j}-m)\right)}^{2}\cdot \sum_{i=1}^{X}\sum_{\;j=1}^{Y}((I_{i,j}-m)\cdot {\left(M_{i,j}-m)\right)}^{2}}}$	The picture width and height, denoted by X and Y, respectively, and the mean value of the reference model M, denoted by *m*, are computed together with an *i*.	ND	*ND*	ND	Leo et al. (44)
	DA	$DA\;(t)=\;\;ZO(t)\cdot \left(1-\frac{1-CO\;(t)+PR(t)+DE(t)}{k_{1}}\right)$	DA is based on the four components *Zone* (ZO), *Control* (CO), *Pressure* (PR), and *Density* (DE), where the first two components increase, and the last two components decrease its value	ND	*ND*	ND	Link et al. (41)
	PPM	$\overset{S}{\underset{s=1}{\sum }}(1-y_{risk}^{s})\overset{U}{\underset{u=1}{\sum }}(y_{risk}^{s}-1)\cdot$	*S* and *U* are the number of successful and unsuccessful passes	ND	*ND*	ND	Power et al. (74)
	DP%	$\frac{\sum_{i=1}^{n}i=DPS}{\sum_{i=1}^{n}i=DPA}$	DPS is the number of difficult passes completed and DPA is the number of difficult passes attempted. This measure shows which players can pass and complete the most risky/difficult passes	ND	*ND*	ND	Power et al. (74)
	PRA	PRA = 1−X D_pr_	X D_pr_ is the probability of a pass being completed	ND	*ND*	ND	Power et al. (74)
	TPA	TPA = PPM + PRA	TPA shows which players help their team keep or lose the ball more than an average player	ND	*ND*	ND	Power et al. (74)

AI, artificial intelligence; ANN, artificial neural networks; DA, dangerousity; BiLSTM, bi-directional long short-term memory; BPI, ball possession intervals; CNN, convolutional neural network; DistEn, distribution entropy; DT, decision tree; DTMC, discrete-time Markov chain; EM, expectation maximization; GB, gradient boosting; HMMs, hidden Markov models; ITFPS, intelligent team formation and player selection; KC, Kolmogorov complexity; LDA, linear discriminant analysis; LSTM, long short-term memory network; LR, logistic regression; ML, machine learning; MLR, multinomial logistic regression; MPOs, moving point objects; MSE, mean squared error; ND, not described; NRME, normalized root-mean-square error; PCA, principal component analysis; PPM, passing plus minus; PRA, passes received added; QDA, quadratic discriminant analysis classifier; QTC, qualitative trajectory calculus; RF, random forest; RIPPER, repeated incremental pruning produce error reduction; SDR, system of distinct representatives; SEn, shannon entropy; SVM, support vector machines; TAI, total activity index; TPA, total passes added; TS, time series; UMAP, uniform manifold approximation and projection; VRNN, variational recurrent neural networks; xG, expected goals.

**Table 3:** Sample characteristics and testing methodologies of the included studies.

Reference	Dataset	Season	Competition	Sample (*N*)	Sampling	Platform	Publisher	QS
Clijmans et al. (31)	Spatiotemporal data	2019−2020	EPL	380 matches	ND	SPADL	Springer	0.68
Chawla et al. (32)	Spatiotemporal data	2007–2008	EPL	2,932 passes	10 Hz	STATS LLC	ACM^a^	0.81
Cho et al. (33)	Technical-tactical KPI	2017–2018	5 leagues	ND	ND	ND	IJSSC^b^	0.83
Bialkowski et al. (34)	Spatiotemporal data	ND	ND	380 matches	ND	Prozone	IEEE^c^	0.85
Bialkowski et al. (35)	Spatiotemporal data	ND	ND	380 matches	ND	Prozone	IEEE^c^	0.86
Beernaerts et al. (36)	Spatiotemporal data	ND	ND	144,086 movements	25 Hz	VBMA	PlosOne	0.90
Bravo et al. (61)	Technical-tactical KPI	2019	La Liga	65,505 actions	ND	InStat API	SDU^d^	0.75
Brooks et al. (50)	Technical-tactical KPI	2012–2013		18,000 passes	ND	Opta Sports	ASA^e^	0.70
Decroos et al. (51)	Technical-tactical KPI	2017–2019	EPL	400,000 actions	ND	Statsbomb	LNAI^f^	0.65
Fernando et al. (59)	Spatiotemporal data	ND	ND	380 matches	ND	Prozone	ACM^a^	0.76
Forcher et al. (52)	Spatiotemporal data	2020/2022	Bundesliga	153 matches	5–2 5Hz	TRACAB	Sci Med Footb	0.82
Garcia-Aliaga et al. (38)	Technical-tactical KPI	2012–2019	18 leagues	50,000 matches	ND	Opta Sports	IJSSC^b^	0.92
Garcia-Aliaga et al. (53)	Technical-tactical KPI	2014–2018	4 leagues	>600 matches	ND	Opta Sports	Taylor and Francis	0.88
Goes et al. (42)	Spatiotemporal data	2017–2018	Eredivisie	16,943 passes	10 Hz	STATS LLC	BigData	0.79
Goes et al. (13)	Spatiotemporal data	ND	Eredivisie	302 matches	10 Hz	STATS LLC	IMA^g^	0.75
Gu et al. (43)	Spatiotemporal data	2019–2020	EPL	43 matches	ND	ND	J Knosys^h^	0.81
Gudmundsson and Wolle (40)	Spatiotemporal data and KPI	ND	ND	ND	ND	SportsCode	JCompenvurbsys	0.79
Leo et al. (44)	Spatiotemporal data	2006–2007	Italian Serie A	16.500 views	ND	VBMA	ACM^a^	0.68
Link et al. (41)	Spatiotemporal data	2014–2015	Bundesliga	64 matches	ND	TRACAB	PlosOne	0.82
Lucey et al. (45)	Spatiotemporal data	2020–2021	EPL	34 matches	ND	Opta Sports	ACM^a^	0.75
Kim et al. (54)	Spatiotemporal data	2019–2020	South Korean	750 matches	10Hz	GPS data	Springer	0.66
Kusmakar et al. (47)	Spatiotemporal data and technical-tactical KPI	2018–2019	MLS	13 matches	ND	ND	IEEE^c^	0.83
Narizuka and Yamazaki (55)	Spatiotemporal data	2016–2017	J1 League	45 matches	ND	J1 player tracking data	Scientific reports	0.88
Nouraie et al. (72)	Spatiotemporal data	2014–2022	EPL	152 matches	ND	GPS data	Springer	0.90
Ötting and Karlis (73)	Spatiotemporal data	ND	ND	ND	25 Hz	Metrica sports	Springer	0.86
Pappalardo et al. (48)	Spatiotemporal data	2015–2016	18 leagues	19,619 matches	ND	Whyscout	ACM^a^	0.81
Power et al. (74)	Spatiotemporal data	2014–2016	EPL	726 matches	10 Hz	EPL player tracking data	ACM^a^	0.85
Shen et al. (37)	Spatiotemporal data	2021–2022	WCL	10 matches	ND	VBMA	Scientific reports	0.89
(49)	Spatiotemporal data	2015–2016	ND	ND	25 Hz	STATS SportVU	arXiv	0.65
Stival et al. (70)	Spatiotemporal data	ND	Brazilian Serie A	10 matches	30 Hz	DVideo	PlosOne	0.86
Yücebaş (56)	Spatiotemporal data	2010/2014	FIFA WC	ND	ND	FIFA player tracking data	Springer	0.71

EPL, English premier league; J1, Japan league 1; KPI, key indicators performance; MLS, major league soccer (EUA); QS, quality assessment; VBMA, video-based motion analysis; WC, world cup; WCL, UEFA women's champions league.

^a^ ACM transactions on spatial algorithms and systems.

^b^ *IJSSC International Journal of Sports Science and Coaching*.

^c^ *IEEE Transactions on Knowledge and Data Engineering*.

^d^ *Statistical Analysis and Data Mining*.

^e^ *The ASA Data Science Journal*.

^f^ *Lecture Notes in Computer Science.*

^g^ *IMA Journal of Management Mathematics.*

^h^ *Knowledge-Based System*.

**Table 4:** Key outcomes of the reviewed articles according to study purpose, tactical analysis, AI methods, data visualization, and data processing.

Reference	Study purpose	Tactical analysis	AI	Data visualization	Data processing	Key outcomes
Clijmans et al. (31)	Analyzing the offensive playing style of teams is an important task within soccer analytics that has various applications in match preparation and scouting	Playing style	ST	Sequential patterns of a team's style and offensive style	DTMC	The model allows for a generalization of a team's past behavior, and the extracted style is less influenced by the rarity of shots and goals. Capture a team's positional and sequential style, as well as reason about the style's efficiency and similarities with other teams
Chawla et al. (32)	Automating the evaluation of passes in soccer matches using trajectory data	Trajectory signals and computational geometry	ML	Computational geometry and passing rating	Player motion model	Achieved 90.2% accuracy in pass rating, demonstrating the effectiveness of the model. Used complex data structures from computational geometry to compute predictor variables for pass rating
Cho et al. (33)	Analyze the player's pass style with enhanced accuracy using the deep learning technique	Passing style descriptor	DL	Pass2vec	Convolutional autoencoder	Pass2ve charactered player's styles with improved accuracy will enable us to understand passing better for player training and recruitment
Bialkowski et al. (34)	Introducing an unsupervised method for learning formation templates from tracking data in soccer	Team formation	ML	Large-scale team analysis	Unsupervised ML	Provided insights into team formations and dynamics using tracking data. Developed a method for aligning tracking data at the frame level and identifying team structures
Bialkowski et al. (35)	Determine the identity of a team from spatiotemporal player tracking data	Team style	ML	K-means	Unsupervised ML	This approach characterizes individual team behavior significantly better (3 times more) than other match descriptors that are normally used to describe team behavior using a time-series analysis and predictions approach
Beernaerts et al. (36)	Explore a spatiotemporal qualitative calculus describing the relative spatial movement pattern recognition	Spatial movement pattern	ML	QTC-matrix sequence representation	QTC	Basics of the QTC method for spatial movement pattern recognition such as players perform specific spatial movement patterns when playing against other specific players
Bravo et al. (61)	Modeling techniques to explore and categorize the data and use it to evaluate the types of plays that are most often correlated with victories	Individual and team sports analytics	ML	Offensive and defensive attack plots	xG ECOM	Game data to produce offensive and defensive attack plot from individual player actions
Brooks et al. (50)	Analyzing pass event data in soccer and passing styles based on pass locations	Team pass locations	ML	Analysis of pass locations and patterns	Player ranking	Teams are characterized by passing styles based on pass locations; Ranked players by the value of their passes
Decroos et al. (51)	Proposes SoccerMix, a soft clustering technique based on mixture models for football actions	Typical behaviors of teams and players	ML	Soft clustering technique (SoccerMix)	Probabilistic representation for soccer actions	Overcame sparsity of event stream data; Identified team and player playing styles; Provided alternative view on team's style focusing on opponent response
Fernando et al. (59)	Explored goal-scoring patterns in soccer using player and ball tracking data	Goal-scoring patterns	ND	Fine-grained tracking data from Prozone	Clustering multiagent trajectories EGV model	Utilized clustering of multiagent trajectories and EGV model for analysis. Identified and quantified goal-scoring methods of teams and compared goal-scoring styles
Forcher et al. (52)	Predict ball gains in defence using tracking data to identify tactical variables that drive defensive success	Defensive behaviors	ML	Predict successful (ball gain) vs. unsuccessful defensive plays (no ball gain)	LR, XGBoost, RF classifier	Identify tactical principles of defensive play that appear to be related to gaining the ball: press the ball leading player, create numerical superiority in areas close to the ball (press short pass options)
Garcia-Aliaga et al. (38)	Determine the on-field playing positions of a group of football players based on their technical-tactical behavior	In-game behavior	ML	Riemann geometry algebraic topology	t-SNE UMAP RIPPER	AI-based tactical behavior enriches the understanding of the behavior profiles and game patterns of the different positions
García-Aliaga et al. (53)	Identify differences between leagues and teams, determining the most discriminating variables were obtained as a set of rules discovered	Offensive, defensive, and buildup behavior	ML	OPTA's on-ball event records	t-SNE RIPPER	The evolution of playing styles has meant that teams in the major European leagues seem to be approaching the homogeneity of technical-tactical behavior, determined by fewer free kicks, fewer long passes but more vertical and more errors in ball control but greater success in dribbling
Goes et al. (42)	Quantify pass effectiveness in soccer using tracking data, focusing on disrupting opposing defences	Pass effectiveness	ML	Developed novel measures for evaluating pass effectiveness based on tracking data	Tacking data Traditional data	Provided insights into tactical performance and pass effectiveness in soccer matches
Goes et al. (13)	Assess tactical performance by abstracting a set of spatiotemporal features from the general offensive principles of play in soccer using position tracking data	Tactical performance Match outcome	ML	Scoring opportunity heatmap	DT, GB, LDA, and QDA classifiers	Using only position tracking data, we can provide valuable feedback to coaches about how their team is executing the various principles of play, and how these principles are contributing to overall tactical performance and match outcome prediction
Gu et al. (43)	Quantify team space-control efficiency in given in-game possession using an ML approach	In-game space-control efficiency	ML ANN	Possession model Pitch Control map	CNN LSTM	The created model is used to measure how effectively a team's tactical behavior and positional dynamics align with optimal performance metrics, such as pass completion, player positioning, and spatial control. The model's enhanced performance using a deep generative modeling on picture datasets and contextual elements for conditioning during prediction
Gudmundsson and Wolle (40)	Analyze a football match is without doubt an important task for coaches, talent scouts, players and even the media; and with current technologies	Players movement	ML	Global and local correlation	Clustering correlation	Reports on the most common movement (geographically) patterns formed by the ball transported between defense and offense, as well as player movement patterns
Leo et al. (44)	Develop a multiview system able to understand in real time the interactions between the ball and the players	3D ball trajectories	ML	Virtual Playfield	Correlation	This model allows to identify who is kicking the ball and to establish the exact moment of the engagement from Italian first division soccer championship was used to test the method, and results of the system's performance
Link et al. (41)	Develop models for detecting individual and team ball possession in soccer using position data	Ball possession and control	ML	Ball possession types and control based	Real-time quantification	Automated event detection and provided insights into match dynamics using Bundesliga data
Lucey et al. (45)	Developed a method to estimate chances in soccer using strategic features from player and ball tracking data	Shot efficiency and team strategy	TS	Strategic features’ impact on goal likelihood	LR conditional random field	Analyzed spatiotemporal patterns before shots from a full season of tracking data. Quantified shot efficiency and team strategy based on spatiotemporal data analysis
Kim et al. (54)	Develop a multiview system able to understand in real time the interactions between the ball and the players	3D ball trajectories	TS	Real-time Multiview analysis	6MapNet	Models hold great promise for the development of an automated system for complicated event identification, such as offside violation, where it is crucial to precisely locate the players on the field and identify the frame in which the shot was made.
Kusmakar et al. (47)	Quantify team's performance derived using player interactions into events ending with a goal attempt, that is, ‘‘Shot’’	Dynamical team performance	ML	Coarse-grain player interaction model	TAI SEI KCI DistEn SVM	The study demonstrates that the analysis we present can help uncover the pattern dynamics of a team's network derived using possession chain data, by quantitatively analyzing measures of performance that have a specific distribution and that can be used to predict the performance of a team
Narizuk (55)	Understanding factors influencing player performance across different positions on the pitch	Team formations	ML	Team formations and transition patterns	Clustering algorithm Delaunay method	Identification of average formations and specific patterns; Extraction of team styles and transition patterns from multiple games
Nouraie et al. (72)	Assisting football coaches in making informed decisions about team formation and player selection using ITFPS	Team formation Player selection	ANN ML	ITFPS	Ratio metric	ITFPS generates decisions that are on a par with those made by coaches who are successful. This strategy improves team performance by making the most of team formations and allowing coaches to use rotating formations
Ötting and Karlis (73)	Compute hidden HMMs to analyze high-frequency tracking data, where the underlying states serve for a team's tactic	Space control Playing space	ML	EPS	HMMs	Using copula-based HMMs to compute the EPS of both teams to account for the competitive nature of the game. Our model thus provides an estimate of a team's playing style at each time point
Pappalardo et al. (48)	Design a data-driven framework multidimensional and role-aware evaluation of the performance of football players	Data-driven performance evaluation and player ranking	ML	PlayeRank	Player versatility and efficiency	Player Rank is a valuable tool to support professional football scouts in evaluating, searching, ranking, and recommending football players. Also, it can be easily extended by making individual performance and spatiotemporal trajectories
Power et al. (74)	Addressing the limitation of binary pass evaluation in soccer by estimating risk and reward dimensions using tracking data	Estimated risk and reward of passes	ML	Deployed passing system	Binary labels 0 (unsuccessful) or 1 (successful)	Developed methods for estimating risk and reward dimensions of passes. Provided a nuanced understanding of passing quality and its implications for team performance
Shen et al. (37)	Explores a CNN-based method based on CNN, hoping to provide accurate training feedback in women's football teams	Accurate training feedback	ANN	Convolution layer Activation function Pooling layer Fully connected layer	CNN	The application of CNN helps to provide more accurate evaluation results. The statistics of players’ personal mistakes based on CNN not only provides a real-time analysis tool for the coach team but also helps the coach to formulate coping strategies more quickly
Shokrollahi et al. (49)	Extract player position time-series data to build a competent model for representing team tactics behavioral patterns and predict the outcome of arbitrary movements	Player time series analysis	ML ANN	Players and team movements	Robust Kernel Fuzzy and deep CNN	A hybrid of fuzzy and deep convolutional neural networks for the multivariate time series and dimensional space. The data are extracted from the sport VU player tracking dataset using “Play-by-play dataset” during the match
Stival et al. (70)	Analyze the first five seconds after one team gains ball control to determine if the ball will reach the final quarter of the field and create a goal-scoring chance	Goal-scoring chance	ML	Modeling players’ interactions	FETW Deep CNN	Identifying that offensive play near the adversary penalty area can be predicted by analyzing the first five seconds after ball possession. Revealing the main metrics contributing to the prediction outcome, providing explainability to the models used for soccer match analysis
Yücebaş (56)	Analyze the individual performances according to pitch positions from FIFA World Cups through multilayer ANN	Team performance analysis	ANN	Establishing multi-layer ANN for dataset	Determining architecture and hyper-parameters	Factors affecting player performances ranked by Gedeon's relative importance calculation; visualization of player performance on different pitch positions

AI, artificial intelligence; ANN, artificial neural networks; BiLSTM, bidirectional long short-term memory; BPI, ball possession intervals; CNN, convolutional neural network; DistEn, distribution entropy; DL, deep learning; DT, decision tree; DTMC, discrete-time Markov chain; EGV expected goal value; EPS, effective playing space; FETW, feature extraction time-window; GB, gradient boosting; HMMs, hidden Markov models; KCI, Kolmogorov complexity; ITFPS, intelligent team formation and player selection; LDA, linear discriminant analysis; LSTM, long short-term memory network; LR, logistic regression; ML, machine learning; ND, not described; QDA, quadratic discriminant analysis; RIPPER, repeated incremental pruning produce error reduction; RF, random forest; SEn, Shannon entropy; STGNN, space-time graph neural network; SVM, support vector machines; TAI, total activity index; TS, time series; TC, qualitative trajectory calculus; UMAP, uniform manifold approximation and projection; VRNN, variational recurrent neural networks; xG, expected goals.

This information represents a diverse array of techniques and metrics used in AI fields, specifically ANN, DL, ML, TS, and KPIs. The ANN methods included convolutional neural networks (CNNs), long short-term memory (LSTM), recurrent neural networks (RNNs), variational recurrent neural networks (VRNNs), and variational autoencoders (VAEs). In addition, they encompassed methodologies like the Delaunay method, player rank, hierarchical clustering, logistic regression (LR), XGBoost, random forest (RF) Classifier, and repeated incremental pruning produce error reduction (RIPPER), and dimensionality reduction techniques, such as principal component analysis (PCA) and T-distributed stochastic neighbor embedding (t-SNE). Furthermore, they cover graph theory metrics such as Betweenness centrality, eccentricity, efficiency, vulnerability, clustering coefficient, and page rank, along with performance indicators like expected possession value (EPV) and pitch control map classifier and various others, including computer vision techniques, expected goals (xG), 3D ball trajectories, dangerousity assessment (DA), pass probability model (PPM), and total passes attempted (TPA).

Table 2 displays the measure, formula, description, and AI-based procedures of the reviewed articles. Figure 1 expresses the clustered research hot topic using AI to map tactical behaviors, collective behavior, and movement patterns in football.

## 3 Results

### 3.1 Search findings

**Figure 2:**
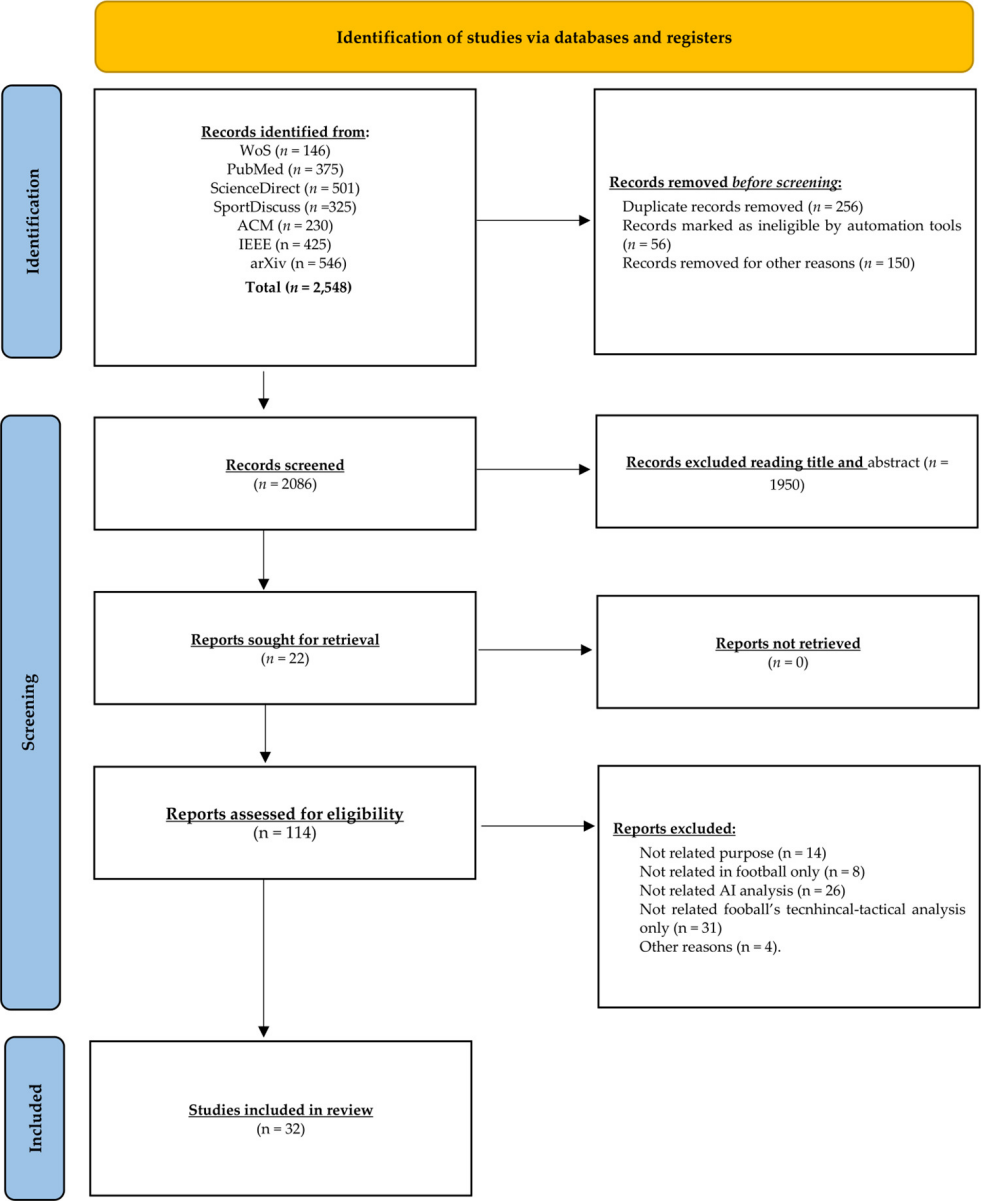
PRISMA flowchart with search results. ACM, ACM Digital Library; AI, Artificial Intelligence; arXiv, arXiv.org (e-Print archive); IEEE, IEEE Transactions on Knowledge and Data Engineering; WoS, Web of Science (Core Collection: Citation Indexes).

A total of 2,548 titles were collected on three academic databases (WoS = 146; Pub-Med = 375, ScienceDirect = 501, and SportDiscus = 325) and digital libraries (ACM = 230; IEEE = 425; arXiv = 546). After applying the selection criteria, 114 full-text articles were screened for eligibility, and 32 articles were retained for a final review. Figure 2 shows an s-PRISMA flow diagram depicting the screening procedures and search results.

### 3.2 Participant characteristics

Table 3 shows the participants’ characteristics of the reviewed studies. Spatiotemporal data (*n* = 20) and technical-tactical KPI (*n* = 7) were the datasets of the AI-based analyses present in the studies. Also, three studies use both datasets concomitantly. The seasons analyzed the period ranging from 2006 to 2007 and then from 2007 to 2021–2022, which means that 16 years were analyzed. English Premier League (EPL) was the most representative league using AI-based analysis (*n* = 9). Bundesliga and Eredivise were represented by two studies each. Other leagues with a single-reviewed study include La Liga, Serie A, Women's Champions League, FIFA World Cup (WC), and Brazilian Serie A (*n* = 6). Three studies analyzed datasets from 5 to 18 leagues in professional contexts and were not based exclusively on one team and/or league. Five studies did not describe the competition level or sporting season of their samples. The sample sizes across the included studies varied, ranging from as few as 2,932 passes to as high as 400,000 actions. Various sampling rates included 10, 25, and 30 Hz; however, 16 studies did not report sampling frequencies from tracking systems. Multiple tracking data, VBMA, and KPI platforms were utilized for data collection and analysis, among which are SPADL (*n* = 1), STATS LLC (*n* = 3), not described (ND) (*n* = 3), Prozone (*n* = 3), VBMA (*n* = 2), InStat API (*n* = 1), Opta Sports (*n* = 3), Statsbomb (*n* = 1), SportsCode (*n* = 1), TRACAB (*n* = 2), GPS data (*n* = 2), Japan League (J1) player tracking data (*n* = 1), Metrica Sports (*n* = 1), Whyscout (*n* = 1), EPL player tracking data (*n* = 1), STATS SportVU (*n* = 1), DVideo (*n* = 1), and FIFA player tracking data (*n* = 1). Various publishers included ACM (*n* = 4), ASA (*n* = 4), BigData (*n* = 1), IJSSC (*n* = 2), IEEE (*n* = 3), LNAI (*n* = 1), PlosOne (*n* = 1), Sci Med Footb (*n* = 1), Scientific Reports (*n* = 2), SDU (*n* = 1), Springer (*n* = 3), arXiv (*n* = 2), ND (*n* = 3), and Taylor and Francis (*n* *=* *1)*. QS scores ranged from 0.65 to 0.92, indicating a moderate to high methodological quality among the included studies.

### 3.3 Quality assessment

The quality assessment (QA) scores in the dataset ranged from a minimum of 0.65 to a maximum of 0.92. The mean score, calculated by summing all values and dividing by the total number of studies, was approximately 0.798, indicating an overall moderate to high methodological quality among the included studies.

### 3.4 Data extraction

Table 4 presents a summary of football data analysis studies, outlining the methods, tactical insights, and key outcomes. Sixteen studies utilized ML techniques for technical-tactical tasks such as pass evaluation, team formation analysis, player performance evaluation, space-control efficiency quantification, and predicting defensive success. Nine studies on the analysis of player and ball tracking data to derive insights into various aspects of football, including shot efficiency, team strategy, defensive behaviors, and pass effectiveness. Three studies mentioned data visualization techniques such as real-time quantification, plotting offensive and defensive attack plots, and visualizing player performance on different pitch positions. Ten studies mentioned employed advanced techniques like classic ANN, DL such as CNN, LSTM networks, and RNN such as accurate training feedback, player TS analysis, modeling players’ interactions, and predicting offensive plays. Among these are each with its own unique set of measures, formulas, and training algorithms. For instance, the calculation of Eigenvalues at specific positions in CNNs involves intricate formulas that account for convolution kernels and input feature maps, while training often relies on unsupervised learning approaches, yielding impressive accuracies ranging from 88% to 94%. Four studies focused on evaluating player and team performance using data-driven approaches, role-aware evaluation, estimating risk and reward dimensions of passes, and multidimensional evaluation of player performance. Tactical analysis was a common theme in all included studies, including the evaluation of passes, quantifying team space-control efficiency, determining team formations, coordination transition patterns, and analyzing player interactions. The analyses would vary from a micro (individual), meso (group), and macro (sector or collective) level.

## 4 Discussion

This systematic review examines and maps out the current state of research on AI-based tactical behavior and collective dynamics in football. The reviewed research analytics have employed various AI techniques to delve into the intricacies of football performance at both individual and team levels, concretely ANN, DL, ML, KPI, and TS techniques. Concretely, the AI algorithms reviewed were the CNN, RNN, VRNN, LSTM, and VAE. They also include techniques such as XGBoost, RF Classifier, PlayerRank, hierarchical clustering, LR, Delaunay method, RIPPER, and dimensionality reduction techniques (PCA, t-SNE). Furthermore, they cover graph theory metrics such as betweenness centrality, eccentricity, efficiency, vulnerability, clustering coefficient, and page rank, along with performance indicators like EPV and pitch control map classifier and various others, including computer vision techniques, xG, 3D ball trajectories, DA, PPM, and TPA. The technical-tactical KPI was expressed by team possession, team formation, team strategy, team space-control, team formations, coordination patterns, analyzing player interactions, ball trajectories, and pass effectiveness.

In the realm of performance tactical analysis utilizing AI-based algorithms, passing style becomes a defining descriptor, dictating the rhythm and flow of the game. Team formation serves as the canvas upon which strategies are crafted. Each team exhibits a unique style, manifested through spatial movement patterns and the nuanced behaviors of both individual players and the collective dynamics. Within this tapestry of play, goal-scoring patterns reveal themselves, defensive behaviors take shape, and in-game behaviors offer valuable insights. Pass effectiveness becomes a crucial metric, measuring a team's tactical performance and influencing the ultimate match outcome. Space-control efficiency transforms into a battleground, where teams compete for dominance over playing space, leveraging data-driven insights to evaluate performance and rank players accordingly. Amid the game's dynamic nature, the risk and reward of passes are constantly assessed, providing accurate training feedback and informing strategic decisions. Player TS analysis offers a deeper understanding of individual performance, while the pursuit of goal-scoring chances drives comprehensive team performance analysis. In this ever-evolving and non-linear dynamic landscape, team formations shift playing positions, with each movement influencing the trajectory of the game. Also, the trajectories of 3D balls carve through space, revealing the dynamism of team performance and the classified types of ball possession and control. Shot efficiency becomes a cornerstone of team strategy as players navigate the complex interplay between technical skill and tactical opportunity. Ultimately, this tactical dynamic transcends the boundaries of the game, offering a glimpse into the intricate world of sports analytics, where spatiotemporal data and data analytics converge to unlock the secrets of victory.

In fact, football's data, research, practitioners, and analysts have been delving deep into the intricate dynamics of the game, seeking to unveil patterns, styles, and strategies that underlie the sport's essence. Among these endeavors, Clijmans et al. (31) undertook a meticulous examination of an offensive playing style, recognizing its paramount importance in match preparation and scouting endeavors. Their work delved into the realm of sequential patterns of a team's style and offensive style, employing a discrete-time Markov chain (DTMC) model to generalize the past behaviors of teams. This model aimed to extract styles less influenced by the rarity of shots and goals, thereby capturing both the positional and the sequential dimensions of a team's style. In addition, it allowed for the evaluation of style efficiency and similarities with other teams, enriching the understanding of football tactics. In addition, Chawla et al. (32), pioneered the automation of pass evaluation in football matches, leveraging trajectory data and computational geometry. Through the application of ML techniques, particularly a player motion model, this model achieved a remarkable 90.2% accuracy in pass rating. Their methodology, rooted in complex data structures derived from computational geometry, paved the way for a more nuanced understanding of passing dynamics within the game. Meanwhile, Cho et al. (33) ventured into the realm of deep learning techniques to analyze player pass styles with heightened precision. This innovative approach utilized passing style. The descriptor, utilizing a convolutional autoencoder under the moniker Pass2vec, aimed to characterize player styles with enhanced accuracy. By doing so, the researchers envisioned facilitating a better understanding of passing dynamics, thereby potentially revolutionizing player training and recruitment strategies.

In a complementary effort, Bialkowski et al. (34) aimed to identify a team's tactical “signature” by analyzing spatiotemporal player tracking data, employing ML techniques focused on the detection of collective positioning patterns. Leveraging unsupervised ML techniques such as K-means clustering, this study devised an approach that significantly outperformed conventional match descriptors in characterizing team behavior. Their work, focusing on TS analysis and predictive modeling, illuminated the distinctive styles and strategies adopted by different teams, thereby enriching the understanding of dynamic coordination. Another study by Bialkowski et al. introduced an unsupervised method aimed at learning formation templates from spatiotemporal tracking data in football. Their approach, rooted in ML principles, enabled large-scale team analysis by providing insights into team formations and dynamics (35). By aligning spatiotemporal tracking data at the frame level to identify team collective structures and patterns, their methodology contributed to a deeper understanding of the strategic nuances inherent in playing style. Beernaerts et al. (36) focused on spatial movement patterns utilizing a multilayer ANN to analyze individual tactical performances across different playing positions. This approach introduced a qualitative trajectory calculus, known as QTC, to recognize these tactical patterns, offering a nuanced understanding of player dynamics on the field. Shen et al. (37) proposed a CNN-based method aimed at providing accurate training feedback in women's football teams. By employing CNN architecture, they developed real-time analysis tools for coaches, enhancing evaluation precision and facilitating quicker strategy formulation. García-Aliaga et al. (38) delved into determining on-field playing positions based on technical-tactical behavior using ML algorithms, enriching the understanding of player roles and game patterns. In their 2021 study, Goes et al. (39) developed an ML model to assess pass effectiveness in football by analyzing spatiotemporal tracking data, with a particular emphasis on disrupting opposing defenses.

Through the application of ML techniques, the reviewed studies devised novel measures for evaluating pass effectiveness, shedding light on tactical performance and strategic decision-making in football matches (39–42). Otherwise, another project by Goes et al. (42) assessed tactical performance by abstracting spatiotemporal features from general offensive principles of play. Utilizing position tracking data, they employed classifiers such as DT, GB, LDA, and QDA to provide valuable feedback to coaches regarding team execution and overall tactical performance, thereby contributing to match outcome prediction (39, 42). Gu et al. (43) contributed to the field of football analytics by quantifying team space-control efficiency during in-game possession. By employing models like ANN, CNN, and LSTM, they measured space-control effectiveness, enhancing the understanding of team dynamics and strategic decision-making on the field. Thus, it is possible to apply DL models to quantify team space-control efficiency, emphasizing dynamic territorial dominance metrics, although both studies address representations of collective behavior. Meanwhile, Gudmundsson and Wolle (40) analyzed player movement patterns, employing clustering techniques to uncover the most common spatial and temporal formations that emerge during a football match. By examining players and the movement of the ball between defensive and offensive zones, they provided valuable insights into team strategies and tactical implementations for both training and competition (39, 40). In a complementary effort, Leo et al. (44) pioneered the development of a multiview system capable of understanding real-time interactions between the ball and players, utilizing 3D ball trajectories to accurately identify moments of player engagement. Tested on data from Italian first division football championship, their system demonstrated promising potential for automated event identification, particularly in complex scenarios such as offside violations. Link et al. (41) focused on developing models for detecting individual and team ball possession using position data, providing real-time quantification and insights into match dynamics. Their automated event detection systems, based on Bundesliga data, enriched the understanding of possession-based strategies and tactical implementations. Lucey et al. (45) proposed a method for estimating score chances in football by leveraging strategic features from player and ball tracking data. Using LR and conditional random field models, they analyzed spatiotemporal patterns preceding shots, thereby quantifying shot efficiency and providing data-driven insights into team strategies.

Lastly, Kim et al. (46) contributed to real-time multiview analysis by developing a system capable of understanding interactions between the ball and the players. By focusing on 3D ball trajectories and employing innovative analysis techniques, their model held significant promise for the development of automated systems for event identification, potentially revolutionizing match analysis and decision-making processes. Gu et al. (43) quantified team space-control efficiency during possession using ML, employing advanced models like CNN and LSTM to enhance predictive capabilities. Kusmakar et al. (47) quantified team performance through player interactions leading to goal attempts, revealing pattern dynamics through possession chain data analysis. Pappalardo et al. (48) designed a data-driven framework for evaluating football players’ performance comprehensively, aiding scouts in player assessment and recommendation. Lastly, Shokrollahi et al. (49) extracted player position TS data to model team tactics and predict match outcomes, employing a hybrid approach of fuzzy logic and deep CNNs for multivariate analysis. Collectively, these studies showcase the diverse applications of ML in dissecting football performance, from individual player actions to team strategies and outcomes. Brooks et al. (50) presented two methods for analyzing pass event data in football, demonstrating their effectiveness through application to the 2012–2013 La Liga season. They showed that teams can be distinguished by their passing styles based on where they attempt passes on the pitch, achieving an 87% accuracy in a team classification task using pass location heatmaps. In addition, they investigated the use of pass locations during possessions to predict shots. Furthermore, they used the weights of the predictive model to rank players by the value of their passes. Decroos et al. (51) addressed the challenge of analyzing playing styles in football, proposing SoccerMix, a soft clustering technique based on mixture models. This approach overcomes the sparsity of event stream data by grouping similar actions together in a probabilistic manner, enabling the characterization of both team and player playing styles. Notably, SoccerMix offers an alternative perspective on a team's style, focusing on how it influences opponents’ playing styles. Forcher et al. (52) focused on analyzing defensive performance in football, utilizing tracking data to predict successful ball gains in defense. They derived player and team metrics from tracking data and trained machine learning classifiers to distinguish successful defensive plays from unsuccessful ones. The study identified tactical principles related to gaining possession, such as pressing the ball-leading player and creating numerical superiority in key areas. García-Aliaga et al. (38, 53) utilized ML algorithms to determine the on-field playing positions of football players based on their technical-tactical behavior. By analyzing non-spatiotemporal descriptors computed from match event records, they identified discriminatory variables for player positions using dimensionality reduction techniques and machine learning algorithms like RIPPER. This approach provided valuable insights for enhancing player performance and identifying positions on the field. FatigueNet, a deep learning algorithm for predicting players’ perceived exertion levels from movement data collected during football sessions. By preprocessing raw GPS data and leveraging deep learning techniques, FatigueNet achieved effective prediction of perceived exertion, offering a potential automated and objective fatigue monitoring system for players (54). In their study, Narizuka and Yamazaki (55) delved into the realm of football analytics by focusing on analyzing player performance in relation to different pitch positions. They emphasized the importance of understanding how various factors influence player performance across different areas of the pitch. To achieve this, they developed a novel clustering algorithm based on the Delaunay method, which enables the characterization of team formations dynamically.

By applying this algorithm to datasets from multiple football games, the studies can identify average formations such as “1-4-4-2,” “1-4-1-4-1,” and “1-4-3-3” and further explore specific patterns within each formation. This method allows for visualization, quantitative comparison, and time-series analysis of formations, providing insights into team styles and player positional exchanges. Tuyls et al. provide a comprehensive perspective on the intersection of AI, game theory, and computer vision in football analytics (8). They highlight the immense potential of leveraging these fields to revolutionize the analysis of both individual players’ and coordinated teams’ behaviors in football. Through a review of state-of-the-art techniques, they illustrate how combining AI, game theory, and computer vision enables various analyses, including counterfactual analysis using predictive models and game-theoretic analysis of penalty kicks with statistical learning of player attributes. Their work underscores the transformative impact of football analytics not only on the game itself but also on the broader field of AI research. Player performance is the most important factor that affects match scores. Factors affecting player performance are not the same for all players and vary according to pitch positions. Analyzing these performance factors in relation to pitch positions can help understand which characteristics of players need to be developed to win. Player training can be arranged accordingly, and team tactics can be changed or improved. Although the importance of analyzing the individual performances of players according to pitch positions has been emphasized in various studies, a large amount of data available have made this analysis difficult. Machine learning can be used to overcome this difficulty. However, ML studies in sports mostly focus on score prediction. There is a lack of traditional and ML approaches that examine the effect of individual player performances on game results. In this context, the datasets of the 2010 and 2014 FIFA WC were analyzed through multilayer artificial neural networks. A specific model was established for each dataset by organizing relevant datasets according to year, player positions, and match levels (group–final). The rectifier linear unit was selected as the activation function for each model. Architecture and hyperparameters for each model were determined through grid optimization. The factors affecting player performances were ranked by Gedeon's relative importance calculation. The average performance indicators for the group matches are 81.34% precision, 87% recall, and 0.84 F1 score (38). They begin by examining existing research on image recognition in football and then proceed to develop a novel football image classification model. This model integrates bidirectional LSTM to extract spatial features and capture temporal dynamics inherent in image sequences. Through rigorous simulation analyses, they demonstrate the model's high recognition accuracy and consistent performance in action recognition and classification tasks. Their findings offer valuable insights into injury prevention and personalized skill enhancement in football training. By analyzing datasets related to sports achievements and employing deep learning models, they identify KPI influencing achievements and develop predictive models for accurate prediction. Their study highlights the importance of understanding and predicting sports achievements, offering valuable insights for improving athletic performance and training strategies (42, 56). The Yücebaş (56) delves into the intricate relationship between player performance and match outcomes in football, particularly focusing on how performance factors vary across different pitch positions. Recognizing the importance of understanding these nuances for strategic planning, the study employs advanced machine learning techniques to analyze datasets from the 2010 and 2014 FIFA WC. By establishing specific models tailored to each dataset and utilizing multilayer artificial neural networks, the study aims to uncover the factors influencing player performances and their impact on match outcome.

Novel spatiotemporal-based models on player movement and team formations were developed based on convolutional neural networks and deep learning architectures (4, 26). Their empirical comparison demonstrated the superiority of these kernels and their efficient approximations for clustering tasks in team sports data, effectively addressing limitations found in existing techniques (25, 57). Supervised and unsupervised ML are distinguished primarily by the presence or absence of labeled data during training (58). In supervised learning, the training data are accompanied by labels indicating the class or category of each data point, allowing models to learn from known outcomes and make predictions accordingly. In contrast, unsupervised learning involves datasets without labels, where the objective is to uncover intrinsic structures, groupings, or patterns—often for clustering or dimensionality reduction—without external guidance. Fernando et al. (59) explored goal-scoring patterns in football using player and ball-tracking data. They utilized fine-grained tracking data from Prozone to cluster multiagent trajectories and developed an EGV or xG model for analysis. Their research aimed to identify and quantify goal-scoring methods of teams while comparing their goal-scoring styles. In addition, Lucey et al. (45) developed a method to estimate chances in football by analyzing strategic features extracted from player and ball tracking data. Their study focused on analyzing spatiotemporal patterns before shots using LR and conditional random field analysis. By quantifying shot efficiency and team strategy based on spatiotemporal data analysis, they provided valuable insights into the factors influencing goal likelihood and team performance (41, 60).

In supervised learning, some of the main techniques include LR classification and neural networks. LR models the relationship between a continuous dependent variable and one or more independent variables, aiming to predict numerical values. Classification techniques, on the other hand, categorize data into predefined classes or categories. Common algorithms include decision trees, k-nearest neighbors (KNN), and support vector machine (SVM). Neural networks, inspired by the functioning of the human brain, consist of multiple layers of artificial neurons and can be applied to both regression and classification problems. Popular architectures include convolutional neural networks (CNNs) and RNNs. In unsupervised learning, the main techniques include clustering, dimensionality reduction, and association rules. Clustering group data are based on similarities with common algorithms including k-means, hierarchical clustering, and Gaussian mixture models. Dimensionality reduction techniques aim to reduce the number of variables in a dataset, while preserving as much variability as possible. Common methods include PCA and t-SNE. Association rule mining identifies frequent relationships between different variables in a dataset, with the most known algorithm being apriorism, often used in market analysis and product recommendation systems. These are among the most widely used techniques in supervised and unsupervised machine learning. The choice of methods depends on the specific problem being addressed and the characteristics of the available data.

### 4.1 Practical applications, research limitations, and future research

Dealing with raw data (*x*, *y*, *z*) from GPS, LPM, or VBMA tracking in sports analytics requires robust IT infrastructure capable of handling large volumes of data, processing it efficiently, and extracting meaningful insights. The reviewed studies highlight the growing importance of advanced analytics, particularly in football, to enhance player performance, tactical awareness, and overall team dynamics. Through innovative approaches such as clustering algorithms, ANN, ML, and DL techniques, and the integration of AI, game theory, and computer vision, researchers are uncovering complex movement patterns within player performances, formations, and game strategies. By analyzing factors such as player positioning, team formations, and transitional patterns, these studies aim to provide valuable insights into optimizing player training, refining team tactics, and ultimately improving game outcomes. Furthermore, the use of advanced data processing methods, such as DL algorithms and image recognition techniques, enables the extraction of comprehensive features from highly complex datasets, allowing for an accurate performance assessment and the development of predictive models. By understanding the nuances of player behaviors and game dynamics, coaches and analysts can make informed decisions to enhance training regimens, develop personalized strategies, and maximize player potential. Moreover, the integration of ML not only facilitates the analysis of retrospective performance analysis but also enables real-time monitoring and predictive insight, empowering teams to adapt and strategize dynamically during matches. Overall, these studies underscore the transformative impact of data-driven approaches in football analytics, offering a deeper understanding of player performance, team formations, and game strategies. By harnessing the power of advanced analytics and AI technologies, researchers aim to revolutionize player development, tactical planning, and overall game management. The insights gained from these studies have the potential to reshape the landscape of football association (football) analytics, driving continuous innovation and improvement in player and team performance analysis. Indeed, the AI-based applications football insights offers substantial practical benefits for understanding training sessions progression or adjusting tactical strategies in real-time during match play. By leveraging spatiotemporal tracking data with advanced modeling techniques, sport scientists, coaches, and performance analysts can identify individual and collective patterns, assess team cohesion and match principles, and simulate opponent behaviors, enabling more informed decision-making in both training process and match management. However, the full potential of these applications remains constrained by critical limitations in data accessibility, replicability, and standardization. Current datasets vary widely in sampling frequency, data structure, and proprietary constraints across different leagues and myriad platforms, posing challenges to cross-study comparisons, algorithmic generalization, and consistent and reliable longitudinal data. To address this, it is essential to advocate for the creation of open-access benchmark datasets and the adoption of standardized data collection protocols. These initiatives would not only enhance the reproducibility of AI-driven tactical analyses but also democratize access to cutting-edge tools for clubs, researchers, and federations with limited resources, fostering broader innovation in performance optimization.

In addition, the practical application in understanding football tactical behavior, collective dynamics, and movement patterns through AI enhances the strategic capabilities of coaches, facilitates player development, improves opposition analysis, provides real-time decision support, enables performance prediction, and enhances talent identification processes in football. Concretely, the new insights can be reported for the data science and match analysis departments of football clubs such as (1) tactical insights: by leveraging AI algorithms to analyze vast amounts of match data in football, coaches and analysts can gain deeper insights into the tactical strategies employed by individual and team performances. This includes understanding patterns of play, positional rotations, pressing schemes, and defensive organization. Such insights can inform tactical adjustments during matches and help teams exploit opponent weaknesses; (2) player development: AI-driven analysis allows for a granular examination of individual player performance within the context of team tactics (33, 61). Coaches can identify players’ strengths and areas for improvement, tailor training programs accordingly, and provide targeted feedback to enhance overall team cohesion and performance (47); (3) opponent analysis: AI-powered systems enable a comprehensive scouting and analysis of upcoming opponents. By dissecting the tactical tendencies, formations, and key player behaviors of opponents, teams can develop specific game plans and counterstrategies to maximize their chances of success (38, 47, 50, 53). (4) Real-time decision support: AI tools can provide real-time insights and recommendations to coaches during matches. By continuously analyzing live match data, these systems can offer suggestions for substitutions, tactical adjustments, and set-piece strategies, empowering coaches to make informed decisions under pressure; (5) performance prediction: AI models can be trained to predict match outcomes based on historical data and contextual factors (7, 19). While not infallible, these predictive analytics can help teams assess their chances against specific opponents and adjust their approach accordingly (36, 47); (6) talent identification: AI-driven analysis can aid in the identification and recruitment of talented players (40).

By analyzing player performance metrics, playing styles, and potential, clubs can make more informed decisions when scouting and signing new players, optimizing their recruitment strategies (40, 52). The AI-based approaches prioritize the identification of positional regularities through pattern recognition in tracking and spatiotemporal data, while others focus on the continuous evaluation of effective playing space by modeling dynamic territorial control. This methodological distinction underscores the specific strengths of each technique. ML models generally offer greater interpretability and are well-suited for segmenting known behavioral patterns, whereas DL models exhibit a higher capacity to model complex and evolving phenomena, although at the expense of interpretability. Consequently, a structured comparative analysis suggests that the selection of AI models should be guided by the nature of the tactical behavior being investigated and the degree of model explainability required for practical application in training and competition. Specifically, AI models were employed to extract meaningful tactical indicators from positional data, enabling pattern recognition in team strategies and player behavior. In this context, there is still a need for professional profiles, combining sports science, data analytics, computer science, and coaching expertise. Also, there are still challenges for the real practical application of the ethical regulation of AI-based techniques in football science.

Other repositories or digital libraries consulted in the systematic review comprise the following: Github (https://github.com/) (31), scipy.cluster.hierarchy and scipy.spatial (55), FA software (40), and HalvingGridSearchCV (52). The most widely used API platforms in the literature for collecting tracking data and KPIs were InStat Inc., Opta Sports, Wyscout, STATS, Sec-ondSpectrum, SciSports, and StatsBomb. A future review will be important to distinguish the differences in KPIs and which studies have been carried out with each of the platforms. The most widely used computer languages are MATLAB, Python, and Rstudio. The prompts for each of these environments should be explored further to better understand what impact AI-based algorithms have on data visualization and, specifically, tactical analysis developing consensus statements, guidelines, and recommendations for model transparency, interpretability, and application of complex AI models (i.e., CNN, LSTM, RNN) and explainable AI (XAI) techniques still dubious and needs a more generalized consensus. Thus, the ethical considerations surrounding and their relevance for practitioner trust and adoption should be deepened. In addition, the institutions, umbrella organizations, and federations must address the key ethical issues, including data privacy, potential algorithmic biases, and the responsible use of player tracking data. These reflections are intended to foster a more critical and responsible application of AI in sports contexts for “black box” models.

The expansion to underrepresented populations and football insights in the dataset routines and coaching decision-making still needs to be explored (4, 26). However, the possibility of automated modeling in the context of predicting training outcomes, match running management (19, 62), talent identification (9, 63), injury prevention (64, 65), and pacing strategies (63, 66) in itself leaves future prospects for expanding the results already published by the studies reviewed. However, the authors should prioritize an integrative approach and massify these datasets. There is still an extensive gap to fill in youth (4, 62) and women's (67–69) football, especially in subelite settings, different competition levels, and contextual variability. Finally, the importance of multidisciplinary teams for AI model development and interpretation, bridging the knowledge gap between developers and end users (e.g., coaches) and developing training programs and digital literacy for effective AI use must be underscored. The next DL, ML, and AI-based models must be developed so that we can make decisions based on (1) real-time decision support, performance prediction, and match management; (2) strategic and tactical thinking, training task design and planning, and substitution managing; (3) practical integration of spatiotemporal tracking data into coaching and practice routines. Also, the effect of these models in other areas of coaching and training should be explored, specifically in organizational management and communication.

## 5 Conclusions

This systematic review summarizes the latest trends in the literature on the use of AI-based methodologies to understand individual and collective tactical patterns in football. Utilizing insights from studies on goal-scoring patterns, spatial movement analysis, and performance evaluation through ANN, DL, and ML, coaches can refine training sessions to enhance offensive tactics, defensive strategies, and player development, ultimately improving team performance on the field. Furthermore, AI-based tactical assessment tools provide real-time and predictive analysis capabilities, improving decision-making processes and tactical planning in football training and competition. In conclusion, AI-based models can effectively reshape the landscape of spatiotemporal tracking data into training and practice routines with real-time decision-making support, performance prediction, match management, tactical-strategic thinking, and training task design. Nevertheless, there are still challenges for the real practical application of AI-based techniques, as well as ethical regulation and the formation of professional profiles that combine sports science, data analytics, computer science, and coaching expertise.

## References

[1] Sarmento H Marcelino R Anguera MT CampaniÇo J Matos N LeitÃo JC. Match analysis in football: a systematic review. J Sports Sci. (2014) 32:1831–43. 10.1080/02640414.2014.898852 24787442

[2] Rico-González M Los Arcos A Nakamura FY Moura FA Pino-Ortega J. The use of technology and sampling frequency to measure variables of tactical positioning in team sports: a systematic review. Res Sports Med. (2020) 28:279–92. 10.1080/15438627.2019.1660879 31516016

[3] Sarmento H Clemente FM Araújo D Davids K McRobert A Figueiredo A. What performance analysts need to know about research trends in association football (2012–2016): a systematic review. Sports Med. (2018) 48:799–836. 10.1007/s40279-017-0836-6 29243038

[4] Teixeira JE Forte P Ferraz R Branquinho L Silva AJ Monteiro AM Integrating physical and tactical factors in football using positional data: a systematic review. PeerJ. (2022) 10:e14381. 10.7717/peerj.14381 36405022 PMC9671036

[5] O’Donoghue P. Research Methods for Sports Performance Analysis. London: Routledge (2009). 10.4324/9780203878309

[6] Sampaio J Maçãs V. Measuring tactical behaviour in football. Int J Sports Med. (2012) 33:395–401. 10.1055/s-0031-1301320 22377947

[7] Folgado H Bravo J Pereira P Sampaio J. Towards the use of multidimensional performance indicators in football small-sided games: the effects of pitch orientation. J Sports Sci. (2018) 0:1–8. 10.1080/02640414.2018.154383 30426856

[8] Tuyls K Omidshafiei S Muller P Wang Z Connor J Hennes D Game plan: what AI can do for football, and what football can do for AI. J Artif Intell Res. (2021a) 71:41–88. 10.1613/jair.1.12505

[9] Teixeira JE Forte P Ferraz R Leal M Ribeiro J Silva AJ Monitoring accumulated training and match load in football: a systematic review. Int J Environ Res Public Health. (2021) 18:3906. 10.3390/ijerph18083906 33917802 PMC8068156

[10] Miguel M Oliveira R Brito JP Loureiro N García-Rubio J Ibáñez SJ. External match load in amateur soccer: the influence of match location and championship phase. Healthcare. (2022) 10:594. 10.3390/healthcare10040594 35455772 PMC9030506

[11] Rein R Memmert D. Big data and tactical analysis in elite soccer: future challenges and opportunities for sports science. SpringerPlus. (2016) 5:1410. 10.1186/s40064-016-3108-2 27610328 PMC4996805

[12] Rico-González M Pino-Ortega J Nakamura FY Arruda Moura F Rojas-Valverde D Los Arcos A. Past, present, and future of the technological tracking methods to assess tactical variables in team sports: a systematic review. Proc Inst Mech Eng Pt P J Sports Eng Tech. (2020) 234:281–90. 10.1177/1754337120932023

[13] Goes FR Meerhoff LA Bueno MJO Rodrigues DM Moura FA Brink MS Unlocking the potential of big data to support tactical performance analysis in professional soccer: a systematic review. Eur J Sport Sci. (2021b) 21:481–96. 10.1080/17461391.2020.1747552 32297547

[14] Gonçalves B Marcelino R Torres-Ronda L Torrents C Sampaio J. Effects of emphasising opposition and cooperation on collective movement behaviour during football small-sided games. J Sports Sci. (2016) 34:1346–54. 10.1080/02640414.2016.1143111 26928336

[15] Memmert D Raabe D Schwab S Rein R. A tactical comparison of the 4-2-3-1 and 3-5-2 formation in soccer: a theory-oriented, experimental approach based on positional data in an 11 vs. 11 game set-up. PLoS One. (2019) 14:e0210191. 10.1371/journal.pone.0210191 30699148 PMC6353547

[16] Herold M Kempe M Bauer P Meyer T. Attacking key performance indicators in soccer: current practice and perceptions from the elite to youth academy level. J Sports Sci Med. (2021) 20:158–69. 10.52082/jssm.2021.158 33707999 PMC7919358

[17] Liu T Yang L Chen H Garcia de Alcaraz A. Impact of possession and player position on physical and technical-tactical performance indicators in the Chinese football super league. Front Psychol. (2021) 12:722200. 10.3389/fpsyg.2021.722200 34659035 PMC8511401

[18] Memmert D Rein R. Match analysis, big data and tactics: current trends in elite soccer. Dtsch Z Sportmed. (2018) 69:65–72. 10.5960/dzsm.2018.322

[19] Teixeira JE Leal M Ferraz R Ribeiro J Cachada JM Barbosa TM Effects of match location, quality of opposition and match outcome on match running performance in a Portuguese professional football team. Entropy. (2021) 23:973. 10.3390/e23080973 34441113 PMC8391710

[20] Bradley PS Ade JD. Are current physical match performance metrics in elite soccer fit for purpose or is the adoption of an integrated approach needed? Int J Sports Physiol Perform. (2018) 13:656–64. 10.1123/ijspp.2017-0433 29345547

[21] Marcelino R Sampaio J Amichay G Gonçalves B Couzin ID Nagy M. Collective movement analysis reveals coordination tactics of team players in football matches. Chaos, Soliton Fract. (2020) 138:109831. 10.1016/j.chaos.2020.109831

[22] Clemente F Oliveira R Akyildiz Z Silva R Ceylan H Afonso J Field-based Tests for Soccer Players: Methodological Concerns and Applications (2022).

[23] Clemente FM Couceiro MS Martins FML Mendes R Figueiredo AJ. Measuring tactical behaviour using technological metrics: case study of a football game. Int J Sports Sci Coach. (2013) 8:723–39. 10.1260/1747-9541.8.4.723

[24] Lutz J Memmert D Raabe D Dornberger R Donath L. Wearables for integrative performance and tactic analyses: opportunities, challenges, and future directions. Int J Environ Res Public Health. (2020) 17:59. 10.3390/ijerph17010059 PMC6981928 31861754

[25] Memmert D Raabe D. Data Analytics in Football: Positional Data Collection, Modelling and Analysis. London: Routledge (2018). 10.4324/9781351210164

[26] Teixeira JE Forte P Ferraz R Branquinho L Silva AJ Barbosa TM Methodological procedures for non-linear analyses of physiological and behavioural data in football. In: Ferraz R Neiva H Marinho DA Teixeira JE Forte P Branquinho L, editors. Exercise Physiology. London: IntechOpen (2022). p. 1–25. 10.5772/intechopen.102577

[27] Page MJ McKenzie JE Bossuyt PM Boutron I Hoffmann TC Mulrow CD The PRISMA 2020 statement: an updated guideline for reporting systematic reviews. Br Med J. (2021) 372:n71. 10.1136/bmj.n71 PMC8005924 33782057

[28] Page MJ Moher D Bossuyt PM Boutron I Hoffmann TC Mulrow CD PRISMA 2020 explanation and elaboration: updated guidance and exemplars for reporting systematic reviews. Br Med J. (2021) 372:n160. 10.1136/bmj.n160 33781993 PMC8005925

[29] Downs SH Black N. The feasibility of creating a checklist for the assessment of the methodological quality both of randomised and non-randomised studies of health care interventions. J Epidemiol Community Health. (1998) 52:377–84. 10.1136/jech.52.6.377 9764259 PMC1756728

[30] Arruda H Silva ER Lessa M Proença D Bartholo R. VOSviewer and bibliometrix. J Med Libr Assoc. (n.d.) 110:392–5. 10.5195/jmla.2022.1434 36589296 PMC9782747

[31] Clijmans J Van Roy M Davis J. Looking beyond the past: analyzing the intrinsic playing style of soccer teams. In: Amini M-R Canu S Fischer A Guns T Kralj Novak P Tsoumakas G, editors. in Machine Learning and Knowledge Discovery in Databases. Cham: Springer Nature Switzerland (2023). p. 370–85. 10.1007/978-3-031-26422-1_23

[32] Chawla S Estephan J Gudmundsson J Horton M. Classification of passes in football matches using spatiotemporal data. ACM Trans Spatial Algorithms Syst. (2017) 3:1–30. 10.1145/3105576

[33] Cho H Ryu H Song M. Pass2vec: analyzing soccer players’ passing style using deep learning. Int J Sports Sci Coach. (2022) 17:355–65. 10.1177/17479541211033078

[34] Bialkowski A Lucey P Carr P Matthews I Sridharan S Fookes C. Discovering team structures in soccer from spatiotemporal data. IEEE Trans Knowl Data Eng. (2016) 28:2596–605. 10.1109/TKDE.2016.2581158

[35] Bialkowski A Lucey P Carr P Yue Y Sridharan S Matthews I. Identifying team style in soccer using formations learned from spatiotemporal tracking data. in 2014 IEEE International Conference on Data Mining Workshop (2014). p. 9–14. 10.1109/ICDMW.2014.167

[36] Beernaerts J Baets BD Lenoir M de Weghe NV. Spatial movement pattern recognition in soccer based on relative player movements. PLoS One. (2020) 15:e0227746. 10.1371/journal.pone.0227746 31945108 PMC6964894

[37] Shen L Tan Z Li Z Li Q Jiang G. Tactics analysis and evaluation of women football team based on convolutional neural network. Sci Rep. (2024) 14:255. 10.1038/s41598-023-50056-w 38168541 PMC10761667

[38] García-Aliaga A Marquina M Coterón J Rodríguez-González A Luengo-Sánchez S. In-game behaviour analysis of football players using machine learning techniques based on player statistics. Int J Sports Sci Coach. (2021) 16:148–57. 10.1177/1747954120959762

[39] Goes FR Kempe M van Norel J Lemmink KAPM. Modelling team performance in soccer using tactical features derived from position tracking data. IMA J Manag Math. (2021a) 32:519–33. 10.1093/imaman/dpab006

[40] Gudmundsson J Wolle T. Football analysis using spatio-temporal tools. in Proceedings of the 20th International Conference on Advances in Geographic Information Systems; New York, NY, USA: Association for Computing Machinery (2012). p. 566–9. 10.1145/2424321.2424417

[41] Link D Lang S Seidenschwarz P. Real time quantification of dangerousity in football using spatiotemporal tracking data. PLoS One. (2016) 11:e0168768. 10.1371/journal.pone.0168768 28036407 PMC5201291

[42] Goes FR Kempe M Meerhoff LA Lemmink KAPM. Not every pass can be an assist: a data-driven model to measure pass effectiveness in professional soccer matches. Big Data. (2019) 7:57–70. 10.1089/big.2018.0067 30321059

[43] Gu C De Silva V Caine M. A machine learning framework for quantifying in-game space-control efficiency in football. Knowl Based Syst. (2024) 283:111123. 10.1016/j.knosys.2023.111123

[44] Leo M Mosca N Spagnolo P Mazzeo PL D’Orazio T Distante A. Real-time multiview analysis of soccer matches for understanding interactions between ball and players. in Proceedings of the 2008 International Conference on Content-based image and Video Retrieval; Niagara Falls, Canada: ACM (2008). p. 525–34. 10.1145/1386352.1386419

[45] Lucey P Oliver D Carr P Roth J Matthews I. Assessing team strategy using spatiotemporal data. in Proceedings of the 19th ACM SIGKDD International Conference on Knowledge Discovery and Data Mining; New York, NY, USA: Association for Computing Machinery (2013). p. 1366–74. 10.1145/2487575.2488191

[46] Kim H Kim J Chung D Lee J Yoon J Ko S-K. 6MapNet: representing soccer players from tracking data by a triplet network. In: Brefeld U Davis J Van Haaren J Zimmermann A, editors. in Machine Learning and Data Mining for Sports Analytics. Cham: Springer International Publishing (2022). p. 3–14. 10.1007/978-3-031-02044-5_1

[47] Kusmakar S Shelyag S Zhu Y Dwyer D Gastin P Angelova M. Machine learning enabled team performance analysis in the dynamical environment of soccer. IEEE Access. (2020) 8:90266–79. 10.1109/ACCESS.2020.2992025

[48] Pappalardo L Cintia P Ferragina P Massucco E Pedreschi D Giannotti F. Playerank: data-driven performance evaluation and player ranking in soccer via a machine learning approach. ACM Trans Intell Syst Technol. (2019) 10:1–27. 10.1145/3343172

[49] Shokrollahi O Rouhani B Nobakhti A. Predicting the outcome of team movements; Player time series analysis using fuzzy and deep methods for representation learning (n.d.).

[50] Brooks J Kerr M Guttag J. Using machine learning to draw inferences from pass location data in soccer. Stat Anal Data Min. (2016) 9:338–49. 10.1002/sam.11318

[51] Decroos T Van Roy M Davis, J. Soccermix: representing soccer actions with mixture models. In: Dong Y Ifrim G Mladenić D Saunders C Van Hoecke S, editors. in Machine Learning and Knowledge Discovery in Databases. Applied Data Science and Demo Track. Cham: Springer International Publishing (2021). p. 459–74. 10.1007/978-3-030-67670-4_28

[52] Forcher L Beckmann T Wohak O Romeike C Graf F Altmann S. Prediction of defensive success in elite soccer using machine learning - tactical analysis of defensive play using tracking data and explainable AI. Sci Med Footb. (2023) 0:1–16. 10.1080/24733938.2023.2239766 37477376

[53] García-Aliaga A Marquina Nieto M Coterón J Rodríguez-González A Gil Ares J Refoyo Román I. A longitudinal study on the evolution of the four main football leagues using artificial intelligence: analysis of the differences in English premier league teams. Res Q Exerc Sport. (2023) 94(2):529–37. 10.1080/02701367.2021.2019661 35438618

[54] Kim J Kim H Lee J Lee J Yoon J Ko S-K. A deep learning approach for fatigue prediction in sports using GPS data and rate of perceived exertion. IEEE Access. (2022) 10:103056–64. 10.1109/ACCESS.2022.3205112

[55] Narizuka T Yamazaki Y. Clustering algorithm for formations in football games. Sci Rep. (2019) 9:13172. 10.1038/s41598-019-48623-1 31511542 PMC6739367

[56] Yücebaş SC. A deep learning analysis for the effect of individual player performances on match results. Neural Comput Applic. (2022) 34:12967–84. 10.1007/s00521-022-07178-5

[57] Gerrard B Memmert D Raabe D. Data analytics in football: positional data collection, modelling and analysis. Sport Manage Rev. (2019) 22(4):568–9. 10.1016/j.smr.2019.01.002

[58] Rico-González M Pino-Ortega J Méndez A Clemente F Baca A. Machine learning application in soccer: a systematic review. Biol Sport. (2022) 40:249–63. 10.5114/biolsport.2023.112970 36636183 PMC9806754

[59] Fernando T Wei X Fookes C Sridharan S Lucey P. Discovering Methods of Scoring in Soccer Using Tracking Data (n.d.).

[60] Knauf K Memmert D Brefeld U. Spatio-temporal convolution kernels. Mach Learn. (2016) 102:247–73. 10.1007/s10994-015-5520-1

[61] Bravo A Karba T McWhirter S Nayden B. Analysis of individual player performances and their effect on winning in college soccer. SMU Data Sci Rev. (2021) 5:8. Available at: https://scholar.smu.edu/datasciencereview/vol5/iss1/8

[62] Teixeira JE Branquinho L Ferraz R Leal M Silva AJ Barbosa TM Weekly training load across a standard microcycle in a sub-elite youth football academy: a comparison between starters and non-starters. Int J Environ Res Public Health. (2022) 19:11611. 10.3390/ijerph191811611 36141883 PMC9517031

[63] Teixeira JE Forte P Ferraz R Branquinho L Morgans R Silva AJ Resultant equations for training load monitoring during a standard microcycle in sub-elite youth football: a principal components approach. PeerJ. (2023) 11:e15806. 10.7717/peerj.15806 37554335 PMC10405799

[64] Morgans R Rhodes D Teixeira J Modric T Versic S Oliveira R. Quantification of training load across two competitive seasons in elite senior and youth male soccer players from an English premiership club. Biol Sport. (2023) 40:1197–205. 10.5114/biolsport.2023.126667 37867738 PMC10588577

[65] Morgans R Rhodes D Bezuglov E Etemad O Di Michele R Teixeira J The impact of injury on match running performance following the return to competitive match-play over two consecutive seasons in elite European soccer players. J Phys Educ Sport. (2023) 23:1142–9. 10.7752/jpes.2023.05142

[66] Branquinho L Ferraz R Forte P Teixeira J Neiva H Marinho D Training Load Variations During Small-Sided Games in Soccer: The Influence of Recovery Time (2022).

[67] Fernandes R Brito J Palucci Vieira L Martins A Clemente F Nobari H In-Season internal load and wellness variations in professional women soccer players: comparisons between playing positions and Status. Int J Environ Res Public Health. (2021) 18:12817. 10.3390/ijerph182312817 34886543 PMC8657164

[68] Branquinho L De França E Teixeira J Forte P Ferraz R. Identifying the ideal weekly training load for in-game performance in an elite Brazilian soccer team. Front Physiol. (2024) 15:1341791. 10.3389/fphys.2024.1341791 38505708 PMC10948442

[69] Branquinho L De França E Teixeira JE Paiva E Forte P Thomatieli-Santos RV Relationship between key offensive performance indicators and match running performance in the FIFA women’s world cup 2023. Int J Perf Anal Spor. (2024) 25(3):580–94. 10.1080/24748668.2024.2335460

[70] Stival L Pinto A dos Santos Pinto de Andrade F Pereira Santiago PR Biermann H da Silva Torres R Using machine learning pipeline to predict entry into the attack zone in football. PLoS One. (2023) 18:e0265372. 10.1371/journal.pone.0265372 36652409 PMC9847968

[71] Wang X Guo Y. The intelligent football players' motion recognition system based on convolutional neural network and big data. Heliyon. (2023) 9(11):e22316. 10.1016/j.heliyon.2023.e22316 38053884 PMC10694318

[72] Nouraie M Eslahchi C Baca A. Intelligent team formation and player selection: a data-driven approach for football coaches. Appl Intell. (2023) 53(24):30250–65. 10.1007/s10489-023-05150-x

[73] Ötting M Karlis D. Football tracking data: a copula-based hidden Markov model for classification of tactics in football. Ann Oper Res. (2023) 325(1):167–83. 10.1007/s10479-022-04660-0

[74] Power P Ruiz H Wei X Lucey P. Not all passes are created equal: objectively measuring the risk and reward of passes in soccer from tracking data. In: Proceedings of the 23rd ACM SIGKDD International Conference on Knowledge Discovery and Data Mining (KDD '17). New York, NY: Association for Computing Machinery (2017). p. 1605–13. Available at: https://dl.acm.org/doi/10.1145/3097983.3098051 (Accessed March 29, 2024).

